# Fear of Being Supplanted: Intergroup Competition Over Prototypicality and Identity Threats Within Social Movements

**DOI:** 10.5334/irsp.951

**Published:** 2025-07-02

**Authors:** Pauline Grippa, Laurent Licata

**Affiliations:** 1 Center for Social and Cultural Psychology, Université libre de Bruxelles, BE; 2 CEVIPOL, Université libre de Bruxelles, BE; 3 FNRS, BE

**Keywords:** Intergroup conflicts, social movements, competition over prototypicality, in-group projection model, symbolic threat, prejudice

## Abstract

We investigated reactions to the emergence of supplanting subgroups among members of dominant subgroups of a social movement. Supplanting subgroups are ideologically and strategically distinct from dominant subgroups and attract social recognition from the general public; thus, they could be perceived as competitors for the status of the movement’s prototypical subgroup. Across three experimental studies, we investigated reactions to supplanting subgroups in comparison to allied subgroups within the movement and ideologically opposing groups to the movement. Supplanting subgroups triggered less negative reactions than ideologically opposing groups but more than allied subgroups. Moreover, they triggered similar levels of symbolic and realistic threat and as much (Study 3) or more (Study 2) competition over prototypicality than ideologically opposing groups. Symbolic threat and competition over prototypicality mediated some of the effects of the type of group on intergroup relations. These findings suggest that, along with symbolic threat, competition over prototypicality can play an important role in shaping conflicts within social movements.

Over the past decade, social struggles have multiplied, increasing visibility to various causes. This expansion has also seen the emergence of new subgroups within existing social movements. While these subgroups can enhance the strength and visibility of movements, they could also create divisions and internal conflicts that may impede efforts to achieve social change (McAdam 1999). Understanding these tensions is crucial for fostering cohesion within social movements and enabling social change.

Conflicts within social movements have been explored from various disciplinary perspectives. Sociologists, for instance, have focused on the structural causes of such tensions, both internal and external, that shape social movements (Balser 1997; Kretschmer 2013; McAdam 1999). These studies highlight how disparities in power, resources and organisational structures can contribute to divisions. Meanwhile, social psychologists have approached these issues through the lens of social identity, focusing on how identity dynamics influence in-group conflicts (Sani 2008). For example, Sani and colleagues have investigated schisms—instances where subgroups secede from a parent supraordinate group—within contexts such as the Church of England and political parties (Sani & Pugliese 2008; Sani & Reicher 2000). Schisms, they argue, occur when changes in norms, values, or group identity create a perception of ‘identity subversion’, leading some members to feel that the group’s identity has fundamentally changed or no longer exists.

While the sociological and social-psychological approaches provide valuable insights, existing research primarily focuses on extreme cases of conflict, such as schisms. However, less attention has been paid to the precursors to such divisions: the internal group dynamics that unfold before a schism occurs. To address this gap, this study examines these earlier dynamics by investigating intergroup relations between subgroups within a social movement. We will explore how dominant subgroups of a social movement—those perceived as prototypical of the social movement—react to the emergence of distinct subgroups that attract social recognition from other activists or the broader public, which we call ‘supplanting subgroups’. Specifically, we propose to investigate whether these dynamics might be explained by competition over prototypicality (Grippa & Licata 2025).

We define these supplanting subgroups as ‘emerging subgroups within social movements, ideologically and/or strategically distinct from the dominant subgroup and attracting social recognition from the general public and other activists’. We suggest that dominant subgroups of a social movement, which are typically recognised as prototypical of the social movement and enjoy symbolic and material advantages (Simon & Klandermans 2001; Wenzel et al. 2008), may perceive supplanting subgroups as competitors for their prototypical position because of their distinctiveness and recognition (Grippa & Licata 2025). Along with competition over prototypicality, we investigated whether the perception of symbolic threat (e.g., to norms and values) and realistic threat (e.g., to material resources) could explain these internal tensions.

To explore these dynamics, we first investigated whether dominant subgroups perceive supplanting subgroups negatively through the measure of perceived proximity, intergroup attitudes and cooperation intentions. Second, we examined whether these reactions are driven by specific threats: perceived competition over prototypicality, symbolic and realistic threats. Finally, to test the specificity of reactions to supplanting subgroups, we compared them with two other types of groups: allied subgroups (expected to trigger fewer conflicts) and ideologically opposing groups (expected to provoke stronger conflict).

This study contributes to the literature on intergroup dynamics within social movements by examining how the characteristics of supplanting subgroups—distinctiveness, social recognition and shared superordinate identity—impact intergroup relations.

## The Three Characteristics of Supplanting Subgroups

Supplanting subgroups are characterised by their distinctiveness, their perceived ability to attract social recognition and their specific categorisation as being part of the same social movement as the dominant subgroup while also being distinct from it at the subordinate level. We posit that each of these characteristics could have negative implications for intergroup relations through the perception of competition over prototypicality and of symbolic and realistic threats.

## Perceived Competition Over Prototypicality

The first defining characteristic of supplanting subgroups is their shared membership in a superordinate category, such as a social movement, alongside other subgroups. According to the Ingroup Projection Model (Wenzel et al. 2008), subgroups within a superordinate category (i.e., a dual identity) often project their distinct characteristics onto the superordinate identity. While this projection occurs across all subgroups within the same superordinate category, only some subgroups are usually recognised as the most prototypical representatives of the superordinate identity: the dominant subgroups. Indeed, being acknowledged as the most prototypical subgroup yields symbolic and material benefits: the ability to define norms and values within the superordinate category (Bell et al. 2022; Wenzel et al. 2008) and access to critical material resources (Zald & McCarthy 1979). Tensions may arise when a newly emergent subgroup, distinct from the dominant subgroup, begins to gain increasing recognition as the most prototypical subgroup of a social movement, which is the case of supplanting subgroups.

Research on prototypicality threats provides insight into such scenarios. Danbold et al. (2023) explored how dominant groups may feel threatened by demographic changes, such as immigration, which challenge their status as representatives of the national identity. They define prototypicality threat as a concern among dominant groups that their claim to embody the broader identity (e.g., ‘America’) may be undermined.

While this research sheds light on the dynamics of prototypicality threats, we suggest that this threat may be better understood as the perception of competition over *social recognition as the most prototypical group* by members of dominant subgroups. Indeed, while Danbold and colleagues (2023) proposed the concept of *prototypicality threat*, they failed to apprehend what causes this threat: the perception that immigrants might blur the prototype of America or that immigrants might *become* the prototypical group of America? While both scenarios are plausible, we suggest that when members of dominant subgroups perceive that a distinct subgroup within the superordinate category is *gaining recognition* as the most prototypical, it fosters a perception of competition over prototypicality and negative intergroup relations.

We propose that social recognition is central to this competition over prototypicality for two reasons. First, achieving dominance within a group requires being recognised as prototypical by others. Second, losing this recognition also impacts individuals psychologically, as recognition is a fundamental human need (Honneth 1996), playing a pivotal role in intergroup dynamics and political struggles (De Guissmé & Licata 2017; Licata et al. 2011; Simon 2020).

The perception of competition over prototypicality intersects with the concept of distinctiveness threat, as outlined by Branscombe and colleagues (1999), and with the perception of symbolic threat (Stephan 2013). However, the three are underpinned by different psychological processes. Indeed, perceived distinctiveness threat concerns identity content and may be experienced by both dominant and other subgroups (i.e., supplanting subgroups) when subgroup boundaries become blurred. In the same line, perceived symbolic threat also concerns identity content and can be experienced by every subgroup within a social movement when another subgroup is presenting views or norms that clash with one’s perceptions (Stephan 2013). On the contrary, we argue that perceived competition over prototypicality is primarily a struggle for social recognition and status within a superordinate category and only affects members of the dominant subgroup who fear losing their status. Thus, as such, competition over prototypicality provides a more targeted lens for understanding tensions between dominant and emerging subgroups within social movements.

Along with the perception of competition, we propose to investigate whether the perception of symbolic and realistic threats also plays a role in in-group conflicts.

## Perceived Symbolic and Realistic Threat

Drawing on Integrated Threat Theory (Stephan 2013), which explains intergroup conflict through the perception of two types of threats—symbolic threat and realistic threat—we hypothesise that supplanting subgroups also trigger such perceptions. We suggest that symbolic threats arise when dominant subgroups perceive ideological and strategic differences with emergent subgroups as challenges to their norms, values, or ideologies. Realistic threats, in contrast, pertain to material competition; members of politicised groups may fear that emergent subgroups will claim the limited resources necessary for the in-group’s survival.

Sociological research has long documented these types of conflicts: ideological and strategic disagreements align with perceptions of symbolic threats, while competition for material resources reflects realistic threats (Kretschmer 2013; McAdam 1999). By integrating social-psychological and sociological perspectives, this study investigates how supplanting subgroups differ from allied subgroups and ideologically opposing outgroups in triggering these perceived threats.

We next introduce two other emergent group types and outline our hypotheses about how reactions to them differ from those to supplanting subgroups.

## Three Types of Emergent Groups

**Figure 1:**
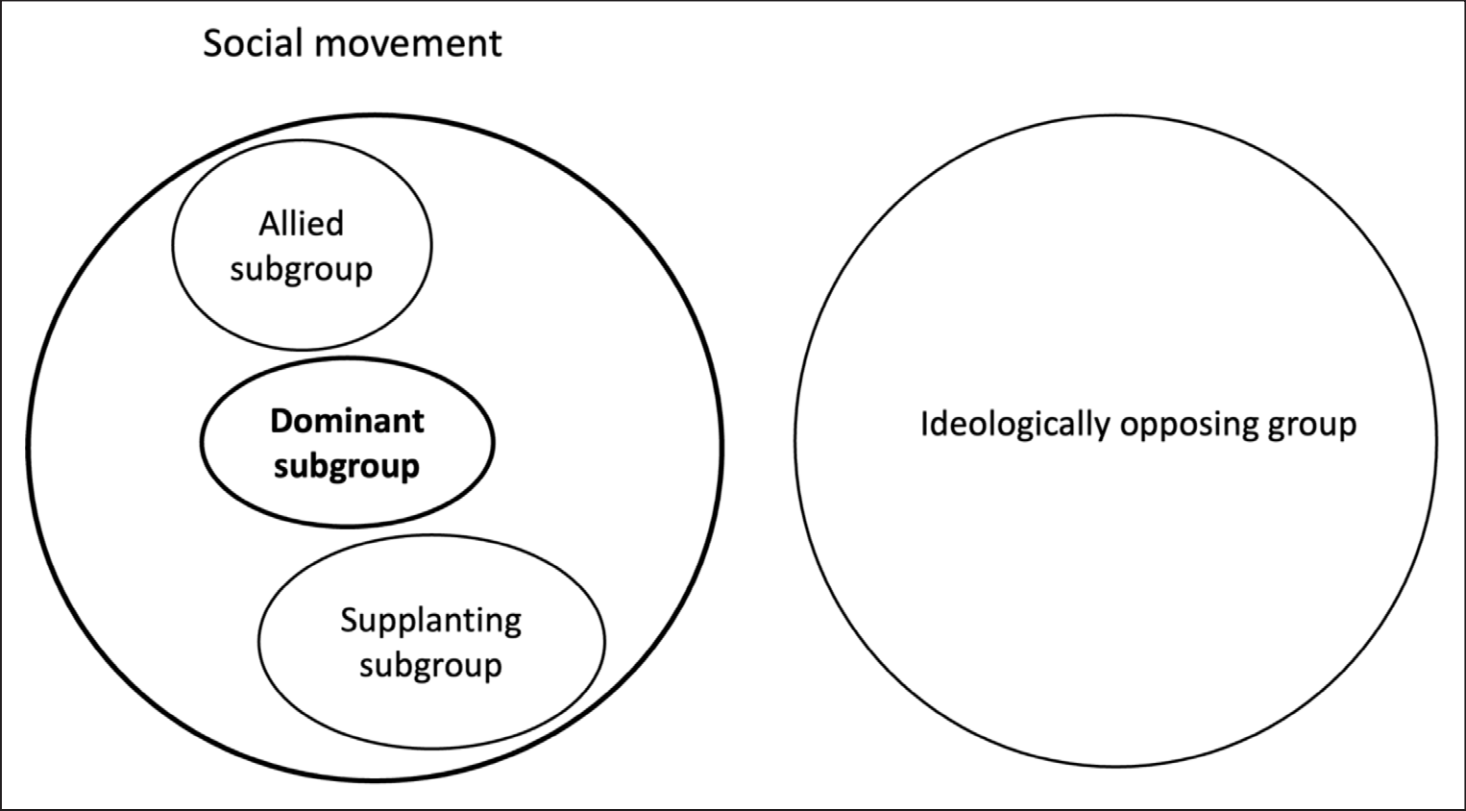
Categorisation of each type of group from the dominant subgroup perspective.

See Table 1 for an overview of the differences between each type of group and Figure 1 for a graphical representation of the categorisation of the three types of groups.

**Table 1:** Differences between the three types of groups.


*TYPE OF GROUP*	DISTINCTIVENESS	ATTRACTION OF SOCIAL RECOGNITION	PART OF THE SOCIAL MOVEMENT

Supplanting subgroup	X	X	X

Allied subgroup			X

Ideologically opposing group	X		


Supplanting subgroups are emergent groups within a social movement that differ ideologically and/or strategically from the dominant subgroup (the in-group) while gaining social recognition. Allied subgroups, by contrast, are also emergent but share ideological and strategic similarities with the dominant subgroup. Although they may differ in dimensions, such as member demographics or time spent within the movement, allied subgroups are characterised by the fact that they share social recognition with the dominant subgroup. Finally, ideologically opposing groups are external to the movement, with ideologies fundamentally incompatible with the overarching values of the social movement. As such, their ideological distinction from the dominant subgroup is greater than that of the supplanting subgroups.

### Hypotheses

**Competition Over Prototypicality**. Supplanting subgroups as part of the same superordinate group and as highly recognised will trigger the perception of competition over prototypicality by members of the dominant subgroup. In contrast, ideologically opposing groups, being outgroups, and allied subgroups, being ideologically and strategically similar and sharing recognition with the dominant subgroup, should evoke less competition over prototypicality.

Moreover, competition over prototypicality will mediate the link between subgroup type and perceived proximity, attitudes and cooperation. We suggest that supplanting subgroups will trigger less perceived proximity, more negative attitudes and less cooperation compared to allied subgroups. On the other hand, while supplanting subgroups may trigger greater competition than ideologically opposing groups, their shared identity with members of dominant subgroups should still lead to less negative reactions than ideologically opposing groups.

**Perceived Symbolic and Realistic Threats**. We propose that both supplanting subgroups and ideologically opposing groups will trigger more perceived symbolic and realistic threats than allied subgroups. However, as supplanting subgroups share the same superordinate category, we expect that members of dominant subgroups will have a stronger in-group bias (Castano et al. 2002), leading to less intense perception of threats compared to ideologically opposing groups. Moreover, we hypothesise that perceived symbolic and realistic threats will also mediate the link between the subgroup type and perceived proximity, attitudes and cooperation.

## Overview of the Studies

**Figure 2:**
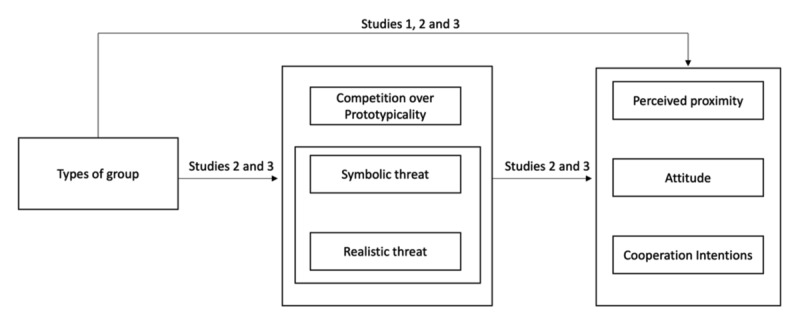
Overview of Hypotheses and Studies.

In Study 1, we analysed the perceived proximity, intergroup attitude and cooperative intentions toward the three types of groups. In Studies 2 and 3, we sought to replicate the results of Study 1 and to test the impact of the type of group on symbolic and realistic threats and competition over prototypicality, as well as the mediation hypotheses (see Figure 2 for an overview of the studies).^1^

In three studies, we used the fictionalised society paradigm to experimentally induce a politicised identity and expose participants to an outgroup. Despite some concerns about external validity, this paradigm has effectively manipulated variables such as economic inequality (Jetten et al. 2015) and conspiracy theories (Bertin 2024) and is well-suited for testing causality in intergroup processes.

Participants were introduced to a role-playing scenario set in the fictional city of Vlurville. They were asked to imagine themselves as engaged citizens leading a thriving and highly recognised group within a social movement, fostering identification with a dominant subgroup (see Supplementary Materials for examples of participants’ engagement with the task). Participants were then randomly assigned to one of three conditions, each depicting the emergence of one of the three groups presented above. Next, participants described their perceptions and emotions toward the new group in a writing task and then completed the survey. Their written responses demonstrated engagement with the task (see Supplementary Materials).

Hypotheses were tested using GLM models in Jamovi. Studies 2 and 3 included parallel mediators, and conditions were compared using contrasts: the first contrast compares supplanting subgroups with ideologically opposing groups (–1, supplanting subgroup; 0, allied subgroup; 1, ideologically opposing group), and the second compares allied subgroups against the other two (–1, supplanting subgroup; –1, ideologically opposing group; 2, allied subgroup). We used contrasts instead of post hoc comparisons, rather than exploring all possible pairwise differences in post hoc analyses.^2^ Even though this approach does not capture the full range of possible differences across conditions, it aligns with our preregistered confirmatory hypotheses and is preferable to post hoc analyses when theory-driven comparisons are at stake because post hoc analyses increase the risk of Type 1 errors. Mediation was verified using Yzerbyt and colleagues’ (2018) guidelines through the test of component paths. Demographic details and descriptive statistics are presented in Tables 2 and 3; correlations are in Table 4.

**Table 2:** Demographic information.


	STUDY 1	STUDY 2	STUDY 3

*N*	198	385	392

Gender			

Women	88.8%	51.9%	51.6%

Men	10.1%	47.7%	46.4%

Non-binary		1 person	

Non-mentioned gender identity	1%	0%	2.1%

Age	19.9 (3.14)	42.1 (12.3)	39 (13.1)

Educational level*			

No degree	6.1%	7.5%	2.8%

High School	87.9%	17.1%	29.2%

Undergraduate	3.5%	42.3%	39.8%

Master	2.5%	29.9%	13.4%

PhD	0%	3.1%	0.8%

Identified as an activist	49.5%	40.5%	24%


*Note*. * Highest diploma obtained.

**Table 3:** Means and standard deviations (Studies 1, 2 and 3).


VARIABLE	STUDY	SUPPLANTING SUBGROUP	ALLIED SUBGROUP	IDEOLOGICALLY OPPOSING GROUP

1. Perceived proximity	1	2.81 (1.18)	3.50 (1)	2.07 (1.25)

	2	2.95 (1.20)	3.60 (1.21)	2.08 (1.35)

	3	3.04 (1.08)	3.77 (0.96)	1.95 (1.09)

2. Intergroup Attitude	1	51.2 (21.7)	78.1 (14)	28.9 (24.7)

	2	51.4 (25.9)	76.3 (20.4)	32.6 (25.6)

	3	57.4 (25.1)	79.1 (19.3)	26.1 (23.8)

3. Cooperation Intentions	1	5.36 (1.29)	6.11 (0.74)	3.41 (1)

	2	4.92 (1.45)	5.96 (0.98)	3.58 (1.13)

	3	5.34 (1.11)	5.70 (0.99)	3.59 (1.14)

4. Competition over prototypicality	2	4.65 (1.51)	3.15 (1.82)	4.18 (1.70)

	3	4.36 (1.61)	2.37 (1.45)	4.24 (1.45)

5. Symbolic Threat	2	4.07 (1.52)	2.63 (1.31)	4.09 (1.55)

	3	3.91 (1.46)	2.39 (1.29)	4.10 (1.40)

6. Realistic Threat	2	4.44 (1.35)	3.28 (1.69)	4.20 (1.67)

	3	4.32 (1.70)	2.62 (1.52)	4.17 (1.65)


**Table 4:** Correlation matrix including all variables in the three studies.


VARIABLE	*STUDY*	1	2	3	4	5	6

1. Perceived Proximity	1	—					

	2	—					

	3	—					

2. Intergroup Attitude	1	.50***	—				

	2	.59***	—				

	3	.77***	—				

3. Cooperation Intentions	1	.50***	.82***	—			

	2	.54***	.74***	—			

	3	.59***	.77***	—			

4. Competition over prototypicality	2	–29***	–.46***	–.44***	—		

	3	–.42***	–.54***	–.43***	—		

5. Symbolic Threat	2	–.31***	–.54***	–.59***	.72***	—	

	3	–.46***	–.62***	–.55***	.73***	—	

6. Realistic Threat	2	–.26***	–.43***	–.41***	.82***	.69***	—

	3	–.36***	–.49***	–.39***	.83***	.69***	—


*Note*. *** p < .001.

## Study 1

### Method

#### Participants

In Study 1, we determined the sample size (*N* = 202) based on a study conducted by Crawford (2014) on intergroup conflicts between ideologically opposing groups. We recruited 210 undergraduate psychology students at a French-speaking Belgian university through the university’s SONA programme with the incentive of extra class credit. After excluding twelve participants (5.7%) for failing attention or seriousness checks, we had a sample of 198 participants, which fell slightly short of our target. A sensitivity analysis revealed that such a sample enables us to detect an effect as small as *f* = .22, which corresponds to a medium to large effect in the social psychology literature (Lovakov & Agadullina 2021).

#### Measures

##### Perceived Proximity

We adapted the identity fusion scale (Swann et al. 2009) to assess perceived proximity between participants’ in-group and the target group. This graphical Likert scale presents five pairs of circles ranging from separate (1) to fully overlapping (5). Participants selected the pair that best represented the perceived proximity.

##### Intergroup Attitude

Participants rated their attitude toward the target group on a thermometer scale (Abelson et al. 1982) ranging from 0 (very negative attitude) to 100 (very positive attitude).

##### Cooperation Intentions

We developed an 8-item scale (α = .89) to assess participants’ intentions to cooperate with the target group (1 ‘*absolutely not*’ to 7 ‘*absolutely*’). For example, we asked, ‘To what extent do you intend to work on a common project with them?’

##### Attention and Seriousness Checks

We assessed participants’ attention and seriousness with two items (see Supplementary Materials for details).^3^

### Results

**Figure 3:**
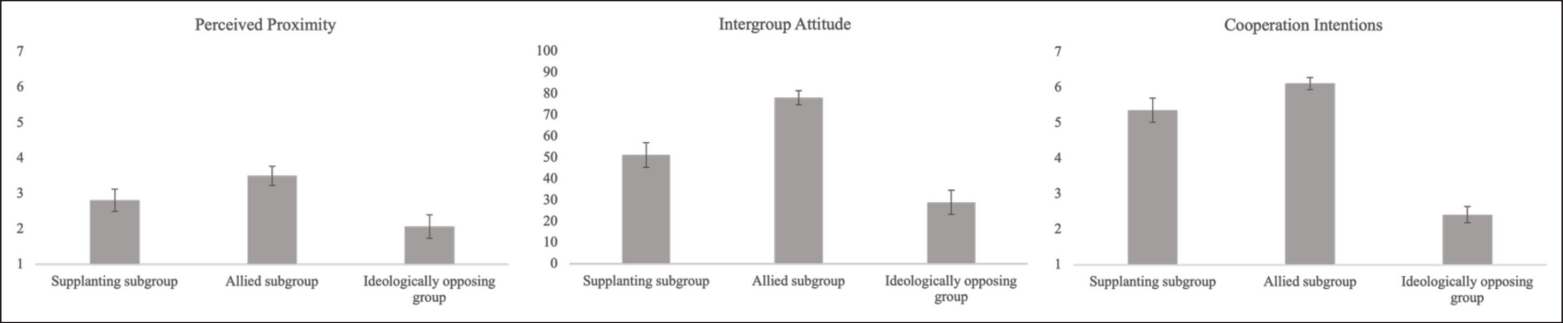
Means of perceived proximity, intergroup attitude and cooperation intentions (Study 1).

See Figure 3 for a graphical representation of our results.

#### Perceived Proximity

In line with our hypothesis, both contrasts were significant with a moderate effect size (b_C1_ = –0.37, 95% CI [–.58; –.17], *t*(195) = –3.63, *p* < .001; b_C2_ = 0.35, 95% CI [0.24; 0.47], *t*(195) = 6.16, *p* < .001), suggesting that supplanting subgroups were perceived to be closer to members of the in-group than ideologically opposing groups but less close than allied subgroups.

#### Intergroup Attitude

Both contrasts were significant with a large effect size (b_C1_ = –11.2, 95% CI [–14.8; –7.52], *t*(195) = –6.04, *p* < .001; b_C2_ = 12.7, 95% CI [10.7; 14.72], *t*(195) = 12.37, *p* < .001). Therefore, as predicted, supplanting subgroups triggered a more positive attitude than ideologically opposing groups and a less positive attitude than allied subgroups.

#### Cooperation Intentions

As predicted, both contrasts were significant with a large effect size (b_C1_ = –0.98, 95% CI [–1.16; –0.80], *t*(195) = –10.8, *p* < .001; b_C2_ = 0.58, 95% CI [0.48, 0.68], *t*(195) = 11.4, *p* < .001), meaning that participants expressed more cooperation intentions toward supplanting subgroups than toward ideologically opposing groups, but less so than toward allied subgroups.

### Discussion

As predicted, supplanting subgroups were perceived as more distant from the in-group, elicited more negative attitudes and prompted lower cooperation intentions than allied subgroups. Conversely, they were seen as closer and provoked more positive responses than ideologically opposing groups. Effect sizes indicated meaningful distinctions in how participants responded to the subgroups. Perceived proximity showed moderate effects, reflecting nuanced perceptions of group closeness, while larger effects on attitudes and cooperation suggested that emotional and behavioural responses were more strongly shaped by subgroup types.

However, our sample—primarily female university students—may limit generalisability due to potential differences in age, education, socioeconomic status and gender (Hanel & Vione, 2016). Additionally, while the sample size was adequate for detecting the main effects, Study 2 tested a more complex model with three mediators. We therefore aimed for a larger, more diverse sample to improve external validity and statistical power. Study 2 also included validated measures of symbolic and realistic threat, as well as competition over prototypicality, enabling us to test additional hypotheses.

## Study 2

### Method

#### Participants

Four hundred and thirteen French speakers participated in the study through the Foule Factory platform in exchange for payment. After excluding 28 participants (6.77%) who failed attention or seriousness checks, 385 participants remained for analysis. We pre-registered the same hypotheses as in Study 1. Our sample size enabled us to detect effects as small as *f* = .15, considered a small to medium effect (Lovakov & Agadullina 2021).

#### Procedure

The same experimental design was used in Study 2. However, we added reliable measures of symbolic and realistic threats and competition over prototypicality.

#### Measures

We used Likert-type scales ranging from 1 [totally disagree] to 7 [totally agree] in all our measures. The items of each scale are available in the Supplementary Materials.

##### Competition Over Prototypicality

Based on De Guissmé and Licata (2017), we adapted a 5-item scale (α = .95) tapping the perception that the in-group lacks recognition because recognition is granted to an outgroup.

##### Symbolic and Realistic Threats

We assessed symbolic threat (α = .77) and realistic threat (α = .90) with three items each, adapted from Green and colleagues (2020).

##### Consequences on Intergroup Relations

Intergroup attitude, cooperation intentions (α = .91) and perceived proximity were measured as in Study 1.

### Results

#### Consequences on Intergroup Relations

**Figure 4:**
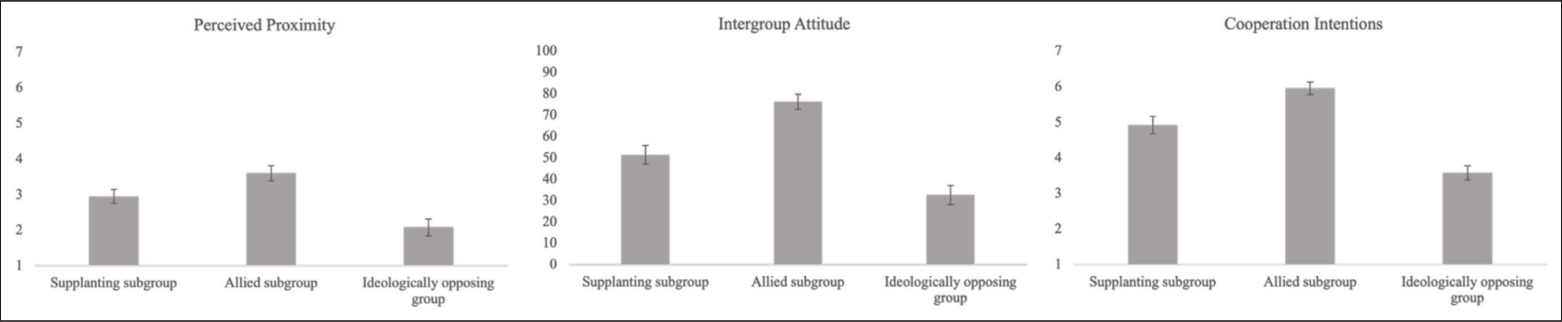
Means of perceived proximity, intergroup attitude and cooperation intentions (Study 2).

See Figure 4 for a graphical representation of our results.

##### Perceived Proximity

As in Study 1 and in line with our hypothesis, both contrasts were significant with moderate effect sizes (b_C1_ = –0.43, 95% CI [–0.59; –0.28], *t*(382) = –5.58, *p* < .001; b_C2_ = 0.36, 95% CI [0.27; 0.45], *t*(382) = 8.01, *p* < .001). Supplanting subgroups were perceived to be closer than ideologically opposing groups, but less so than allied subgroups.

##### Intergroup Attitude

As in Study 1 and in line with our hypothesis, both contrasts were significant with large effect sizes (b_C1_ = –9.44, 95% CI [–12.39; –6.49], *t*(382) = –6.29, *p* < .001; b_C2_ = 11.43, 95% CI [9.72; 13.15], *t*(382) = 13.09, *p* < .001). As predicted, supplanting subgroups triggered a more positive attitude than ideologically opposing groups and a less negative attitude than allied subgroups.

##### Cooperation Intentions

As in Study 1 and in line with our hypothesis, both contrasts were significant with large effect sizes (b_C1_ = –0.67, 95% CI [–0.82; –0.52], *t*(382) = –8.90, *p* < .001; b_C2_ = 0.57, 95% CI [0.48; 0.66], *t*(382) = 13, *p* < .001). Participants expressed more cooperation intentions in the supplanting subgroup condition than in the ideologically opposing group condition but less so than in the allied subgroup condition.

#### Type of threat

##### Competition Over Prototypicality

In line with our hypotheses, both contrasts yielded significant results—moderate for the contrast comparing supplanting and ideologically opposing groups or large for the contrast comparing the allied subgroup with other groups—effect sizes (b_C1_ = –0.23, 95% CI [–0.44; –0.03], *t*(382) = –2.23, *p* < .05; b_C2_ = –0.42, 95% CI [–0.54; –0.30], *t*(382) = –6.97, *p* < .001), meaning that supplanting subgroups elicited significantly more perception of competition over prototypicality than ideologically opposing groups and allied subgroups.

##### Symbolic and Realistic Threats

In line with our predictions, supplanting subgroups triggered more symbolic (b_C2_ = –0.48, 95% CI [–0.59; –0.38], *t*(382) = –9.12, *p* < .001) and realistic (b_C2_ = –0.25, 95% CI [–0.46; –0.23], *t*(382) = –6.10, *p* < .001) threats than allied subgroups with a large effect size. However, contrary to our predictions, supplanting subgroups did not trigger significantly less symbolic (b_C1_ = 0.01, 95% CI [–0.17; 0.19], *t*(382) = 0.12, *p* = .902) and realistic (b_C1_ = –0.12, 95% CI [–0.31; 0.07], *t*(382) = –1.21, *p* = .229) threats than ideologically opposing groups.

#### Mediation analyses

**Figure 5:**
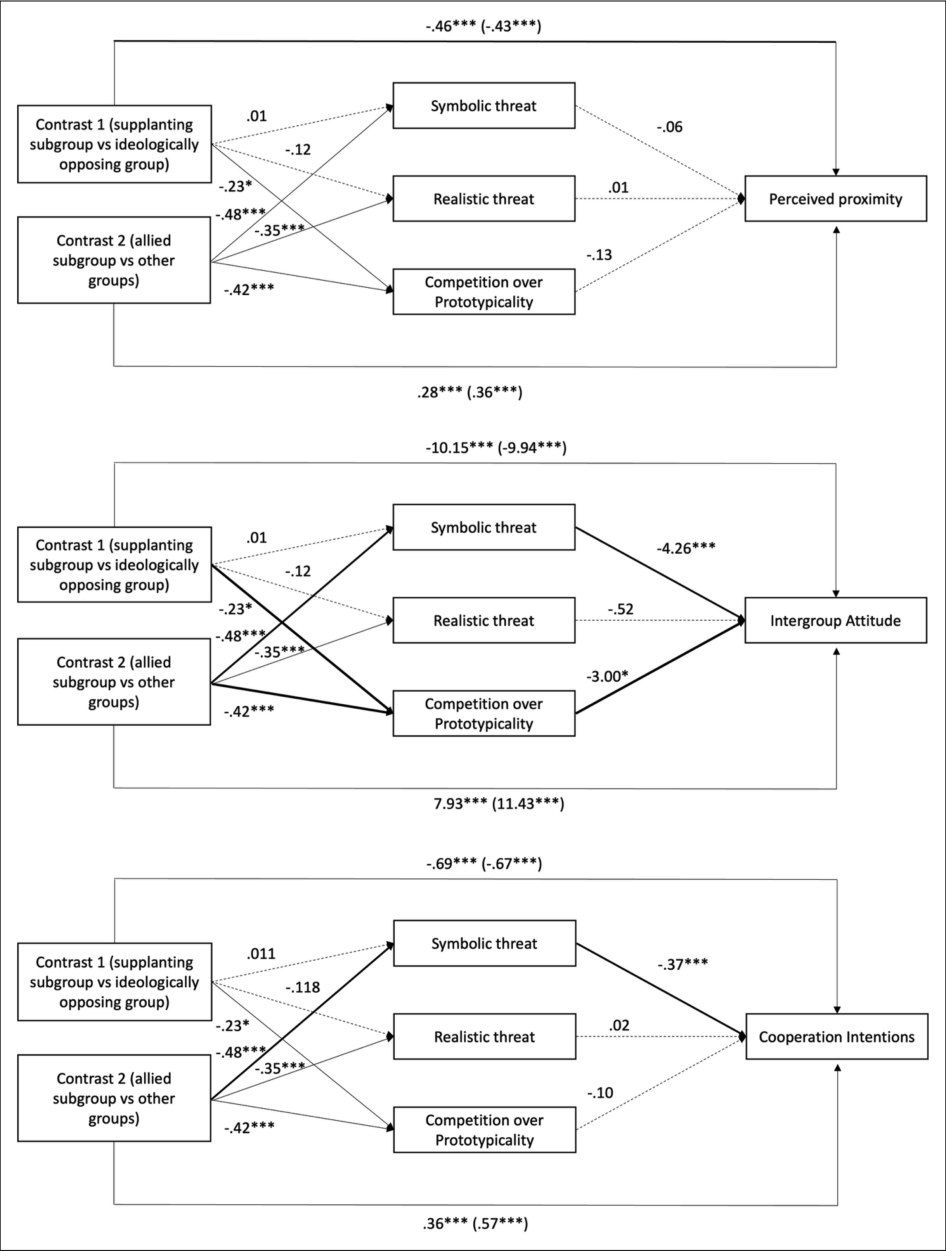
Mediation analysis on perceived proximity, intergroup attitudes and cooperation intentions (Study 2). *Note*. A full arrow indicates that the path is significant. An interrupted arrow indicates that the path is not significant. The bold arrows represent the indirect paths of our mediation hypothesis. Intergroup Attitude was measured using a thermometer scale from 1 to 100.

The detailed results can be found in Figure 5.

##### Perceived proximity

Contrary to our mediation hypotheses, no mediation was found. This means that neither the perception of symbolic and realistic threats nor the perception of competition over prototypicality mediated the impact of the types of groups on perceived proximity.

##### Intergroup Attitude

Competition over prototypicality partially explained negative attitudes toward both supplanting and ideologically opposing groups, as participants perceived greater competition over prototypicality with these groups than with allied subgroups. Moreover, the perception of competition over prototypicality mediated the difference in intergroup attitude between a supplanting and an ideologically opposing group. Additionally, symbolic threat, but not realistic threat, partially mediated the negative attitudes participants had toward both supplanting and ideologically opposing subgroups, compared to allied subgroups. Finally, neither symbolic nor realistic threats mediated the difference in attitudes between supplanting and ideologically opposing groups.

##### Cooperation Intentions

First, the perception of competition over prototypicality did not influence cooperation intentions. Then, symbolic threat partially explained why supplanting and ideologically opposing subgroups reduced cooperation intentions compared to allied subgroups, while realistic threat did not. Finally, neither symbolic nor realistic threats mediated the difference between supplanting and ideologically opposing groups.

### Discussion

Study 2 replicated and extended the findings from Study 1. Supplanting subgroups were perceived more positively than ideologically opposing groups but less positively than allied subgroups. As in Study 1, the type of group had a moderate effect on perceived proximity and a large effect on intergroup attitudes and cooperation intentions, highlighting strong emotional and behavioural reactions to subgroup type. Supplanting subgroups also elicited higher perceptions of realistic and symbolic threats, as well as competition over prototypicality, compared to allied subgroups, with large effect sizes. Compared to ideologically opposing groups, they triggered greater competition over prototypicality (moderate effect), but not greater symbolic or realistic threat.

Mediation analyses showed that symbolic threat partially accounted for the negative attitudes and lower cooperation intentions associated with supplanting and ideologically opposing groups but did not affect perceived proximity. Competition over prototypicality also partially mediated the relationship between group type and intergroup attitudes, but not proximity or cooperation. These partial mediations suggest that while symbolic threat and competition over prototypicality play meaningful roles, other factors also influence intergroup dynamics. This is further discussed in the general discussion.

## Study 3

### Method

#### Procedure

This final study, a direct replication of Study 2, involved participants from the USA, while Study 1 included French-speaking Belgian psychology students, and Study 2 featured French speakers recruited from Foule Factory. The texts and items were translated into English. However, two sentences were changed in the vignettes presenting the supplanting subgroup. For more information, see Supplementary Materials.

#### Participants

In Study 3, 400 participants responded to the study through the Prolific platform in exchange for payment. Eleven of them were excluded due to a failed attention check (*N* = 6) or seriousness check (*N* = 2), leaving 392 participants for analysis (*N* = 392). A sensitivity analysis showed that this sample enabled us to detect an effect size as small as *f* = .15, which corresponds to a small to medium effect size (Lovakov & Agadullina 2021).

#### Measures

All variables were measured with the same scales as in Study 2 and had good or very good internal reliabilities: competition over prototypicality (α = .96), symbolic (α = .71) and realistic threat (α = .92) and cooperation intentions (α = .89).

### Results

#### Consequences on Intergroup Relations

**Figure 6:**
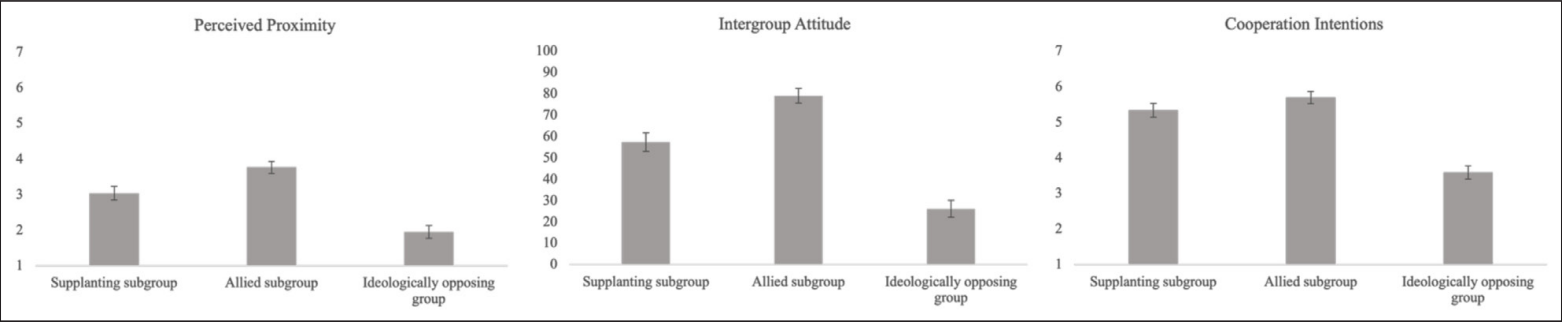
Means of perceived proximity, intergroup attitude and cooperation intentions (Study 3).

See Figure 6, for a graphical representation of our results.

##### Perceived Proximity

In line with our hypothesis and as in Studies 1 and 2, both contrasts were significant with moderate effect sizes (b_C1_ = –0.54, 95% CI [–0.67; –0.42], *t*(389) = –8.46, *p* < .001; b_C2_ = 0.42, 95% CI [0.35; 0.50], *t*(389) = 11.23, *p* < .001). Supplanting subgroups were seen as closer than ideologically opposing groups but less close than allied subgroups.

##### Intergroup Attitude

In line with our hypothesis, and as in Studies 1 and 2, both contrasts were significant with large effect sizes (b_C1_ = –15.7, 95% CI [–18.4; 12.9], *t*(389) = –11.1, *p* < .001; b_C2_ = 12.5, 95% CI [10.8; 14.1], *t*(389) = 15.1, *p* < .001). Supplanting subgroups elicited more favourable attitudes than ideologically opposing groups but less favourable attitudes than allied subgroups.

##### Cooperation Intentions

In line with our hypothesis and as in Studies 1 and 2, both contrasts were significant with large effect sizes (b_C1_ = –0.88, 95% CI [–1.01; –0.74], *t*(389) = –13.2, *p* < .001; b_C2_ = 0.41, 95% CI [0.33; 0.49], *t*(389) = 10.5, *p* < .001). Supplanting subgroups elicited more cooperation intention than ideologically opposing groups but less so than allied subgroups.

#### Type of Threats

##### Competition Over Prototypicality

In line with our hypotheses, the contrast comparing the impact of a supplanting subgroup with an allied subgroup proved significant with a moderate effect, suggesting that supplanting subgroups triggered more competition over prototypicality than allied subgroups (b_C2_ = –0.64, 95% CI [–0.75; –0.53], *t*(389) = –11.33, *p* < .001). However, contrary to our hypotheses and unlike in Study 2, no difference was found between supplanting subgroups and ideologically opposing groups (b_C1_ = –0.06, 95% CI [–0.25; 0.13], *t*(389) = –0.61, *p* = .541).

##### Symbolic and Realistic Threats

Contrary to our hypotheses, and as in Study 2, supplanting subgroups triggered more symbolic (b_C2_ = –0.54, 95% CI [–0.63; –0.44], *t*(389) = –10.73, *p* < .001) and realistic (b_C2_ = –0.54, 95% CI [–0.65; 0.42], *t*(389) = –9.2, *p* < .001) threats than allied subgroups (with a moderate effect size), but no difference was found when compared to ideologically opposing groups (b_C1, symbolic threat_ = 0.09, 95% CI [–0.07; 0.26], *t*(389) = 1.09, *p* = .278; b_C1, realistic threat_ = –0.08, 95% CI [–0.27; 0.12], *t*(389) = –0.76, *p* = .447).

#### Mediation Analyses

**Figure 7:**
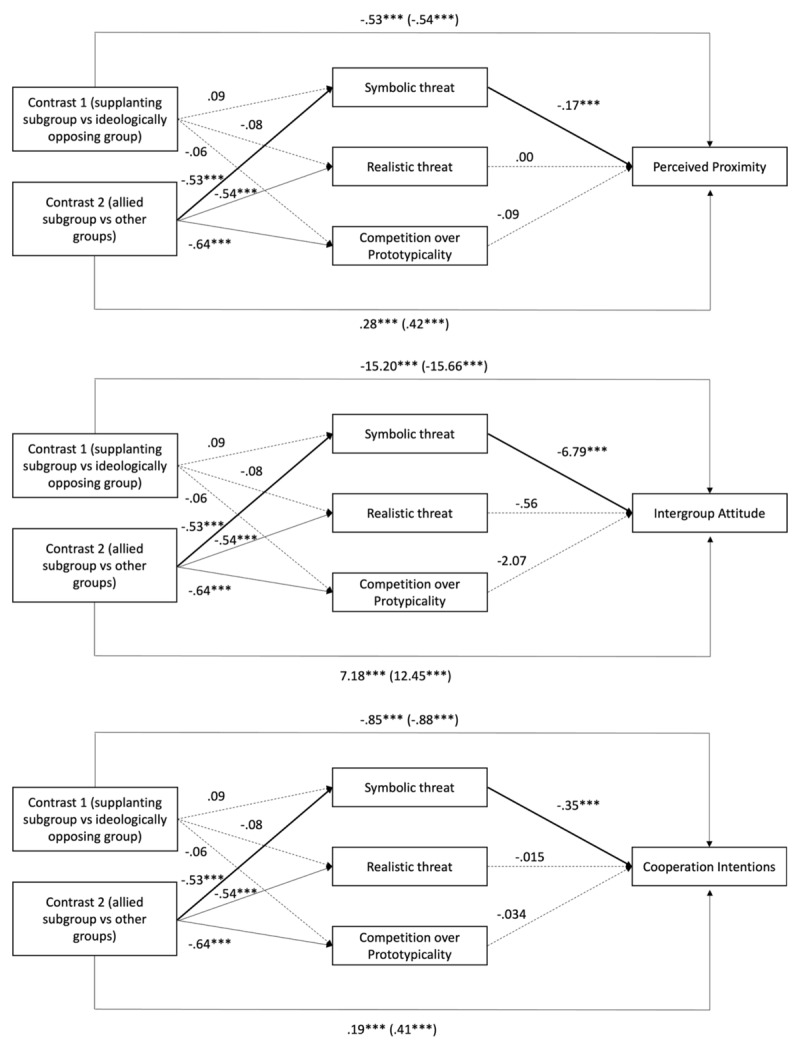
Mediation analysis on perceived proximity, intergroup attitude and cooperation intentions (Study 3). *Note*. A full arrow indicates that the path is significant. An interrupted arrow indicates that the path is not significant. The bold arrows represent the indirect paths of our mediation hypothesis. Intergroup Attitude was measured using a thermometer scale from 1 to 100.

The detailed results can be found in Figure 7.

##### Perceived Proximity

Unlike in Study 2, symbolic threat partially mediated the difference in perceived proximity between allied subgroups and both supplanting subgroups and ideologically opposing groups but did not mediate the difference between supplanting and ideologically opposing groups. Finally, neither realistic threat nor competition over prototypicality mediated the relations between the types of groups and perceived proximity.

##### Intergroup Attitude

First, as in Study 2, symbolic threat mediated the difference in intergroup attitude between allied subgroups and the two other types of groups but did not mediate the difference between supplanting and ideologically opposing groups. Moreover, neither the perception of realistic threat nor competition over prototypicality mediated the relations between Contrast1 or Contrast2 the type of group and intergroup attitude.

##### Cooperation Intentions

The perception of symbolic threat partially mediated the difference between an allied subgroup and the two other groups but not between a supplanting subgroup and an ideologically opposing group. Moreover, neither the perception of a realistic threat nor competition over prototypicality mediated the effects of group type on cooperation intentions.

### Discussion

Study 3 confirmed that supplanting subgroups harmed intergroup relations more than allied subgroups but less than ideologically opposing groups. As in Studies 1 and 2, perceived proximity showed moderate effect sizes, while attitudes and cooperation intentions showed large effects, indicating stronger emotional and behavioural responses than cognitive ones to a subgroup type.

Consistent with Study 2, supplanting subgroups elicited higher symbolic and realistic threats and greater competition over prototypicality than allied subgroups. However, contrary to expectations, supplanting and ideologically opposing groups evoked comparable levels of these threats. This pattern suggests that supplanting groups are seen as challengers to the in-group’s identity and status, triggering responses similar in strength to those typically reserved for ideological outgroups.

Finally, while symbolic threat partially mediated the difference between an allied subgroup and the two other types of groups on outcome variables, no mediation effects were observed for the first contrast comparing a supplanting subgroup with an ideologically opposing group. These findings are further discussed in the general discussion.

## General Discussion

In this research project, our objective was to deepen our understanding of conflicts within social movements. To do so, we examined the reactions of members of dominant subgroups to three types of emergent groups: supplanting subgroups, allied subgroups and ideologically opposing groups. Through three studies, participants were immersed in a fictitious society and imagined themselves as members of a dominant subgroup of a social movement confronted with one of these three groups.

### Impact of Group Type on Intergroup Relations

The three studies consistently supported our hypotheses, showing that supplanting subgroups were perceived as less close, elicited less favourable attitudes and had reduced cooperation intentions compared to allied subgroups, despite belonging to the same movement. However, they were seen as closer, judged more positively and induced greater willingness to cooperate than ideologically opposing groups. These results highlight their unique position as both part of the superordinate in-group and as an outgroup at the subordinate level.

The findings on cooperation intentions carry important practical implications for social movements. Supplanting subgroups elicited lower cooperation intentions than allied subgroups, potentially leading to internal divisions and reduced movement efficacy. Nevertheless, supplanting subgroups still generated a notable willingness to cooperate, greater than that of an ideologically opposing group. This pattern was replicated in a correlational study among feminists, who reported relatively high cooperation intentions with a supplanting subgroup despite perceiving competition over prototypicality (Grippa et al. 2025). This example suggests that cooperation can persist even amid internal competition, indicating that movements may remain effective without perfect unity, provided that overarching goals are clear and widely endorsed.

Second, the findings on perceived proximity offer valuable directions for future research. Although designed to assess perceived distance between groups, this measure also shed light on how participants categorised them: allied subgroups were viewed as part of the in-group (overlapping circles), ideologically opposing groups were viewed as outgroups (separate circles) and supplanting subgroups were viewed as somewhat distinct yet connected (slightly overlapping circles). While informative, this measure serves only as a proxy, as it does not distinguish between common in-group categorisation (i.e., perceiving both groups as one) and dual categorisation (i.e., perceiving them as distinct yet sharing a superordinate identity). This ambiguity leaves open important questions about the role of categorisation: does it moderate the relationship between perceptions of supplanting subgroups and experienced threats or competition, or is it shaped by those perceptions?

Indeed, while we framed the intergroup situation as implying that allied subgroups and supplanting subgroups belong to the same superordinate social category, participants may have perceived it differently. Because of their ambiguous position as part of a common in-group but as outgroups at the subordinate level of social categorisation, supplanting subgroups may trigger different self-categorisation processes among participants, thus changing their reaction towards their members (Turner et al. 1987).

On the one hand, dual categorisation at the subordinate group level could have negative consequences, such as perceptions of competition over prototypicality, because the supplanting subgroup could be perceived as a competing outgroup within the superordinate category (Wenzel et al. 2008). On the other hand, self-categorising at the superordinate level (i.e., the social movement) would include the supplanting subgroup in the common identity and might thus induce positive effects on intergroup relations, as we saw in the results on cooperation intentions (Gaertner & Dovidio 2014).

In our studies, the fact that supplanting subgroups were rated more positively than ideological opponents but less so than allied subgroups may tentatively point to a dual categorisation process being more salient than a common identity process. However, as mentioned before, while perceived proximity could be used as a proxy for self-categorisation, we could not use it as such because it did not allow us to differentiate between two types of categorisation: a dual-identity categorisation and a common-identity categorisation. Thus, this set of studies did not allow us to assess which level of self-categorisation was salient and when. Moreover, we could not investigate the effects of categorisation on perceptions, attitudes and behavioural intentions towards supplanting subgroups, nor the factors that facilitate self-categorisation at one or another level.

Future research could investigate *how* members of dominant subgroups categorise members of supplanting subgroups: either as members of an outgroup, members of a common superordinate group (i.e., common identity) or members of another subgroup sharing a common identity with them (i.e., dual identity). This information would help us understand whether a dual-identity categorisation is linked to more perception of competition over prototypicality, while a common-identity categorisation is linked to less perception of competition.

### Impact of Group Type on Perception of Competition Over Prototypicality and Mediation Analyses

In Studies 2 and 3, supplanting subgroups triggered more perception of competition over prototypicality than allied subgroups. These results suggest that competition over prototypicality can arise within social movements and that specific subgroups, such as supplanting subgroups, trigger more perception of competition over prototypicality than allied subgroups. Moreover, although these results were not entirely replicated in Study 3, we found in Study 2 that a supplanting subgroup triggered more competition over prototypicality than ideologically opposing groups. These results suggest, as hypothesised, that the perception of competition over prototypicality may be more salient when both subgroups share the same superordinate category.

What is more, we found in Study 2 that perceived competition over prototypicality partially mediated the difference in attitude between the different types of groups. These results support our reasoning that, within a social movement, the perception of competition over prototypicality can feed animosity between dominant and supplanting subgroups, even when the perception of symbolic and realistic threats is also considered. However, this partial mediation was not replicated in Study 3, and in Study 2, competition over prototypicality did not mediate the effect of group type on perceived proximity or cooperation intentions.

Differences in sample characteristics may explain some inconsistencies across studies. Study 2 included a higher proportion of self-identified activists, likely increasing participants’ familiarity with intra-movement dynamics and sensitivity to issues of prototypicality. In contrast, the lower activist presence in Study 3 may have reduced engagement with these dynamics. Although in-group identification was not directly measured, this variability suggests it could moderate threat perceptions and reactions. Individuals with stronger activist identification may be more attuned to perceived challenges to the in-group, potentially accounting for the heightened responses observed in Study 2. Future research should directly assess group identification using validated measures (e.g., Leach et al. 2008) and examine its moderating role in shaping emotional, attitudinal, and behavioural responses to intergroup conflict.

Contextual and organisational differences across countries may also shape how competition is perceived. Factors such as resource allocation to social movements may vary between France, Belgium, and North America, influencing the salience of competitive dynamics. Sociological research on factionalism suggests that such dynamics are shaped by both internal structures and external pressures (Balser 1997; McAdam 1999; Kretschmer 2013). Cross-national designs could systematically examine these contextual moderators, including resource scarcity, movement structure, and historical cohesion, by comparing movements with differing internal and external conditions.

### Impact of Group Type on Symbolic and Realistic Threats and Mediation Analyses

The impact of group type on perceived threats is complex. Studies 2 and 3 show that supplanting subgroups trigger higher levels of both symbolic and realistic threats compared to allied subgroups. Moreover, the perception of symbolic threats explains the negative reactions when facing a supplanting subgroup compared to an allied subgroup. Interestingly, we found no mediation effects through the perception of realistic threat, which deviated from our predictions. This suggests that even within a shared social movement, subgroups with different ideologies and/or strategies may be perceived as more threatening, particularly when they attract public attention.

However, when comparing supplanting subgroups to ideologically opposing groups, the results are more mixed. Contrary to our expectations, there was no difference in perceived symbolic threat, indicating that supplanting subgroups are seen as equally threatening as ideologically opposed groups. However, the perception of threat may happen at different levels: while ideological conflict with opposing groups relates to conflicts with an outgroup, ideological conflicts with a supplanting subgroup happen at a subordinate level and relate more to what direction members of both subgroups want the social movement to take. Relatedly, the perception of symbolic threat when facing supplanting subgroups may be more indirect, arising from the perception of competition over prototypicality and questions such as, ‘Who gets to define the ideology of the social movement?’. In this case, individuals may respond by distancing themselves from the rival subgroup, thereby claiming to perceive a symbolic threat to their identity.

Additionally, both supplanting and ideologically opposing subgroups trigger similar levels of realistic threat, likely due to competition for resources. However, the way in which these subgroups threaten access to material resources may differ; ideologically opposing groups could pose a tangible threat by hindering movement growth (e.g., through budget cuts or restrictive laws), while supplanting subgroups could be perceived as replacing the dominant group, depriving them of the resources needed to sustain their activities.

Overall, these results show that symbolic and realistic threats (Stephan 2013) also arise within social movements when members of dominant subgroups face an ideologically and strategically distinct subgroup, at the same level as when they face an ideologically opposing group. Moreover, these results also suggest that perceived symbolic threat, in particular, can trigger conflicts within the movement.

#### Limits

While these studies shed light on the role of psychosocial processes in understanding conflicts within social movements, they present several pitfalls.

First, this series of experimental studies using a fictional scenario paradigm, while allowing us to investigate causal effects by avoiding interference, lacks ecological validity. However, authors have conceptually replicated this study amongst real-life feminists and found that the perception of competition over prototypicality leads to negative attitudes and fewer cooperation intentions (Grippa et al. 2025).

Second, we defined supplanting subgroups as 1) being ideologically and strategically distinct from the dominant subgroup, 2) attracting social recognition and 3) belonging to the same social movement. However, because we decided to fit to real-life situations that a social movement could face, our experimental design failed to disentangle the role of each characteristic of a supplanting subgroup. What is more, while we presented the supplanting subgroup as being ideologically and strategically distinct from the dominant subgroup, we could imagine that both types of distinctiveness could have had a different effect on perceived threats and competition. Further studies could better apprehend the role of ideological versus strategical distinctiveness, as well as the attraction of social recognition and social categorization in explaining conflicts within social movements.

Finally, while we conceptualised the perception of competition over prototypicality as distinct from symbolic and distinctiveness threats in the introduction, we failed to empirically distinguish the perception of competition over prototypicality and from the perception of distinctiveness threat. Indeed, we argued that while symbolic and distinctiveness threats can be perceived by members of different groups and subgroups, competition over prototypicality is specific to dominant subgroups sharing superordinate identities with other subgroups and reflects power struggles and the quest for social recognition. Although our studies aimed to empirically distinguish symbolic threat from competition over prototypicality, we did not measure distinctiveness threat. Therefore, we cannot confirm their empirical distinctiveness. Future research should aim to operationalise distinctiveness threat and test its effects in comparison to competition over prototypicality, perhaps by measuring the perception of distinctiveness threat and conducting exploratory and confirmatory factor analyses to assess the difference between the constructs.

## Conclusion

This project set out to investigate internal conflicts within social movements, with a focus on how members of dominant subgroups respond to emerging ‘supplanting’ subgroups. Across three studies, we compared reactions to supplanting subgroups, allied subgroups, and ideologically opposing groups. The findings showed that dominant subgroup members viewed supplanting subgroups as more distant, held more negative attitudes and were less willing to cooperate compared to allied subgroups. However, supplanting subgroups were seen as closer and induced more positive attitudes and cooperation intentions than ideologically opposing groups.

Participants perceived higher symbolic and realistic threats and competition over prototypicality with supplanting subgroups compared to allied subgroups. Symbolic threat and the perception of competition partially mediated the differences in intergroup attitudes, and only symbolic threat mediated differences in cooperation intentions between allied subgroups and the other groups (specifically, supplanting subgroups). These results suggest that symbolic threat-based conflicts and conflicts around competition appear within social movements.

Moreover, participants perceived slightly more (Study 2) or the same amount of (Study 3) competition over prototypicality when facing supplanting subgroups compared to ideologically opposing groups. In Study 2, perceived competition over prototypicality partially mediated intergroup attitudes, but not proximity nor cooperation intentions, and this mediation was not replicated in Study 3. These results suggest that the perception of competition over prototypicality may be at play between subgroups sharing a superordinate identity and context-dependent.

While these studies contribute to understanding social movement dynamics, the same processes could be observed in other contexts, such as in politics, between scientific disciplines, or in sports fandoms. Further research is needed to replicate these results and deepen our understanding of the underlying social psychological processes, which could help reduce conflicts and improve collective actions.

## Data Accessibility Statement

All studies are preregistered, and data, material and analyses are openly available on the Open Science Framework repository of the projects:

- Study 1: https://osf.io/w6rzy/?view_only=6272731431514fc7bb30dea1174317cd
- Study 2: https://osf.io/h58q6/?view_only=7fc53e89f2594df784ad677686cd3b72
- Study 3: https://osf.io/b4mn8/?view_only=bbd2f7ca28154b51ae6108670dbc0abd
- Data and supplementary materials: https://osf.io/3xr4e/?view_only=335bc1cb0734491a8f0243abe7d845f4

## Additional File

The additional file for this article can be found as follows:

10.5334/irsp.951.s1 Supplementary Materials. Additional analyses, additional measures, experimental material.

## Footnotes

All our hypotheses were preregistered. We preregistered additional measures of warmth and competence but chose to not include them in the main manuscript for clarity. Moreover, for Study 1, we preregistered all the hypotheses, but we chose to only present the results regarding the first three hypotheses, as the threats and competition measures were not optimal.

We conducted Games-Howell post hoc pairwise comparisons as complementary analyses. For more information, see Supplementary Materials.

Our measures can be found in Supplementary Materials. Moreover, we conducted Confirmatory Factor Analyses between perceived threats and perception of competition for Studies 2 and 3 that proved satisfactory (see Supplementary Materials).
