# Supplementary material for: Fear of Being Supplanted: Intergroup Competition Over Prototypicality and Identity Threats Within Social Movements
Source: Int Rev Soc Psychol. 2025 Jul 2;38:8. doi: 10.5334/irsp.951 (PMC12372776; doi:10.5334/irsp.951)
Supplement: Supplementary Materials. — Additional analyses, additional measures, experimental material. [file irsp-38-951-s1.pdf]

Supplementary Materials

Fear of being supplanted: Intergroup competition over recognition within social movements

This document contains supplementary material for studies 1, 2, and 3. More specifically, it contains analyses on the impact of the type of groups on perceived warmth and competence in all three studies. Furthermore, we added the post-hoc comparison analyses mentioned in the manuscript for all three studies, the detailed statistics for mediation analyses in Studies 2 and 3 and the experimental material and measurement scales of all studies.

Study 1

We preregistered hypotheses regarding how warm and competent the three types of groups would be perceived. We hypothesised that a supplanting subgroup would be perceived as less warm and more competent than an allied subgroup, but warmer and less competent than an ideologically opposing group. Contrary to our hypotheses, we found that a supplanting subgroup was perceived as warmer *and* more competent (μ_{warmth}= 3.86, SD_{warmth}= 1.41; $\mu_{competence}$= 5.14, $SD_{competence}$= 1.19) than an ideologically opposing group (μ_{warmth}= 2.80, SD_{warmth}= 1.30; $\mu_{competence}$= 4.08, $SD_{competence}$=1.56), but less warm *and* less competent than an allied subgroup (μ_{warmth}= 5,68, SD_{warmth}= 1; $\mu_{competence}$= 5.80, $SD_{competence}$= .87).

Table 1

Impact of the Type of Group on Perceived Warmth and Competence

	Contrasts									
	Contrast 1 – Supplanting vs Ideologically Opposing Group					Contrast 2 – Allied Subgroup vs Other Groups				
	b	CI Min	CI Max	t-value	*p-value*	b	CI Min	CI Max	t-value	*p-value*
Perceived Warmth	-0.53	-0.75	-0.31	-4.80	<.001	0.78	0.66	0.90	12.74	<.001
Perceived Competence	-0.53	-0.75	-0.31	-4.74	<.001	-0.40	0.27	0.52	6.40	<.001

In Table 2, we report post-hoc comparisons to attest to the difference between each type of group on our outcome variables, especially between a supplanting subgroup and an allied subgroup.

Table 2

Post Hoc Comparisons Study 1

	Supplanting vs Allied		*Supplanting vs Ideologically Opposing*		*Allied vs Ideologically Opposing*	
	t-value	*P-value*	t-value	*p-value*	t-value	*p-value*
Perceived Proximity	-3.41	<.01	3.44	<.01	7.59	<.001
Intergroup Attitude	-7.93	<.001	5.42	<.001	14.84	<.001
Cooperation Intentions	-3.83	<.001	9.27	<.001	18.47	<.001
Perceived Warmth	-8.03	<.001	4.35	<.001	14.97	<.001
Perceived Competence	-3.44	<.01	4.36	<.001	8.26	<.001

Study 2

Method

Measures

Confirmatory Factor Analysis

The results of the CFA between the perceived threats (symbolic and realistic) and perception of competition over social recognition were satisfactory, $\chi^2/df = 3.195$, CFI = .977, RMSEA = .0755 95% CI [.0612, .0902], SRMR = .0344 (Hu & Bentler, 1999[1]), indicating that these three variables could be used independently.

Results

As in Study 1, we preregistered hypotheses regarding how warm and competent the three types of groups would be perceived. However, given the results of Study 1, we hypothesised that a supplanting subgroup would be perceived as warmer and more competent than an ideologically opposing group, but colder and less competent than an allied subgroup**.** According to our hypotheses, and as in Study 1, we found that a supplanting subgroup was perceived as warmer and more competent (μ_{warmth}= 3.93 , SD_{warmth}= 1.37; $\mu_{competence}$= 5.02, $SD_{competence}$= 1.17) than an ideologically opposing group (μ_{warmth}= 3.16, SD_{warmth}= 1.31; $\mu_{competence}$= 4.02, $SD_{competence}$= 1.27), but colder and less competent than an allied subgroup (μ_{warmth}= 5.45, SD_{warmth}= 1.06; $\mu_{competence}$= 5.80, $SD_{competence}$= .89). Statistical details can be found in Table 3.

[1] Hu, L. T., & Bentler, P. M. (1999). Cutoff criteria for fit indexes in covariance structure analysis: Conventional criteria versus new alternatives. *Structural equation modeling: a multidisciplinary journal*, *6*(1), 1-55. https://doi.org/10.1080/10705519909540118

Table 3

Impact of the Type of Group on Perceived Warmth and Competence

	Contrasts									
	Contrast 1 – Supplanting vs Ideologically Opposing Group					Contrast 2 – Allied Subgroup vs Other Groups				
	b	CI Min	CI Max	t-value	*p-value*	b	CI Min	CI Max	t-value	*p-value*
Perceived Warmth	-0.38	-0.54	-0.23	-4.93	<.001	0.63	0.54	0.72	13.96	<.001
Perceived Competence	-0.50	-0.64	-0.36	-7.18	<.001	0.42	0.34	0.50	10.49	<.001

In Table 4, we report Games-Howell post-hoc comparisons to attest to the difference between each type of group on our outcome variables, especially between a supplanting subgroup and an allied subgroup.

Table 4

Post Hoc Comparisons Study 2

	Supplanting vs Allied		*Supplanting vs Ideologically Opposing*		*Allied vs Ideologically Opposing*	
	t-value	*P-value*	t-value	*p-value*	t-value	*p-value*
Perceived Proximity	-4.41	<.001	5.45	<.001	9.36	<.001
Intergroup Attitude	-8.69	<.001	5.90	<.001	14.87	<.001
Cooperation Intentions	-7.87	<.001	8.34	<.001	17.76	<.001
Perceived Warmth	-10.1	<.001	4.62	<.001	15.11	<.001
Perceived Competence	-6.11	<.001	6.54	<.001	12.78	<.001
Perceived Competition over Prototypicality	7.25	<.001	2.32	0.056[2]	-4.64	<.001
Perceived Symbolic Threat	8.26	<.001	-0.12	0.992	-8	<.001
Perceived Realistic Threat	6.12	<.001	1.24	0.432	-4.33	<.001

[2] Contrary to our contrast analysis, the post hoc Games-Howell test did not reveal a significant difference in perceived competition over prototypicality between the supplanting and ideologically opposing groups. However, post hoc pairwise comparisons are generally more conservative and have lower statistical power than planned contrasts, especially when sample sizes are small or group variances are unequal. This increased risk of Type II error (i.e., failing to detect a real effect) may explain why the contrast analysis, which was preregistered and tailored to our theoretical predictions, yielded significant results while the post hoc test did not (Granziol et al., 2025).

Regarding our mediation hypotheses, we found that competition over social recognition mediated both the relations between C1 and perceived warmth and C2 and perceived warmth. This means that a supplanting subgroup will trigger more competition than the two other types of groups, and that this perception of competition is linked with less perception of warmth when facing a supplanting subgroup.

Moreover, we found that symbolic threat mediated the relations between C2 and perceived warmth, meaning that participants perceived the allied subgroup as triggering less symbolic threat than the two other groups and therefore perceived the allied subgroup as warmer than the two other groups. These results corroborate our findings, according to which a supplanting subgroup may also trigger ideological conflicts within social movements.

Figure 1

Mediation Analyses on Perceived Warmth in Study 2

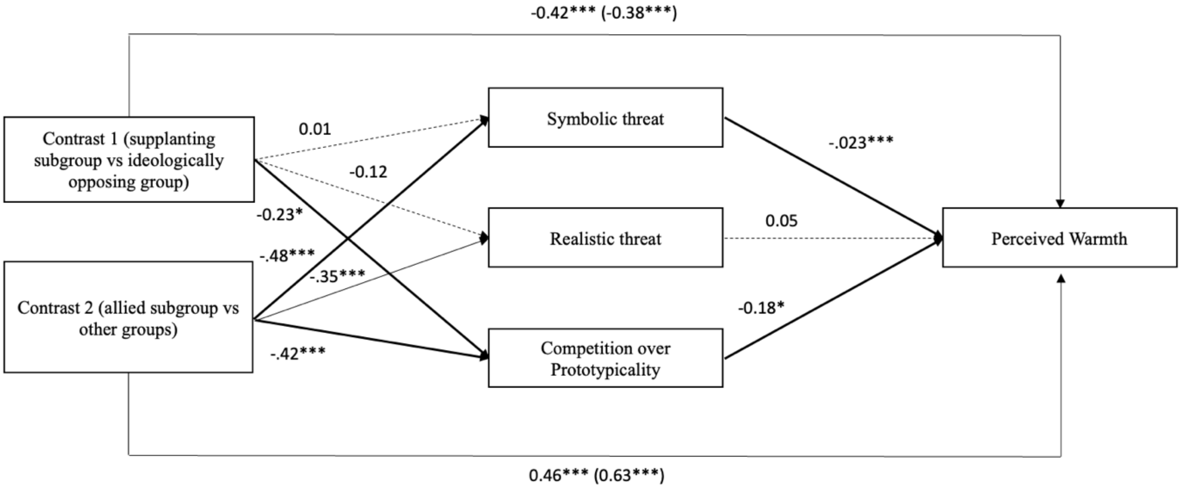

Note. A full arrow indicates that the path is significant. An interrupted arrow indicates that the path is not significant. A full, bold arrow indicates that both paths are significant and that mediation was found significant.

Regarding our mediation hypotheses on the perception of competence, we found that symbolic threat mediated the relations between C2 and the perception of competence. This suggests that, as for the perception of warmth, an allied subgroup was linked to less perception of symbolic threat, which led to more perception of competence than the two other

groups. These results corroborate our findings, according to which a supplanting subgroup may also trigger ideological conflicts within social movements.

Figure 2

Mediation Analyses on Perceived Competence in Study 2

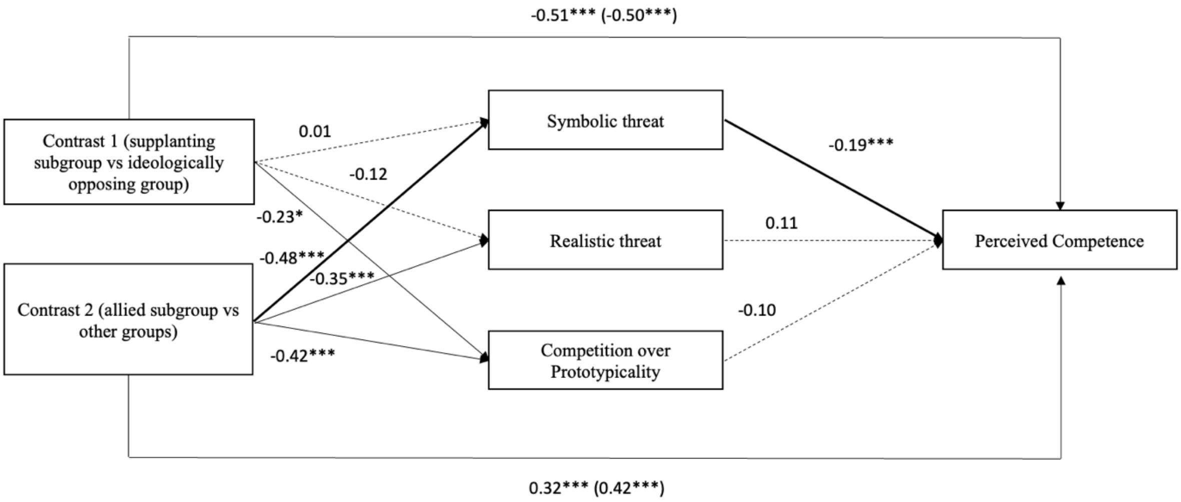

Note. A full arrow indicates that the path is significant. An interrupted arrow indicates that the path is not significant. A full, bold arrow indicates that both paths are significant and that mediation was found significant.

In Tables 5 to 9, we report the detailed statistics of mediation analyses for each dependent variable.

Table 5

Mediation Analyses on Perceived Proximity

Type	Effect	b	95% CI		β	p
			Lower	Upper		
Indirect	Contrast 1 (supplanting vs ideologically opposing) → Competition → Perceived Proximity	0.03	-0.01	0.07	0.02	.142
	Contrast 1 → Symbolic Threat → Perceived Proximity	$-6.47^{e}-4$	-0.01	0.01	$-3.82^{e}-4$	.903
	Contrast 1 → Realistic Threat → Perceived Proximity	0.00	-0.01	0.02	$8.96^{e}-4$	.850
	Contrast 2 (allied vs other groups) → Competition → Perceived Proximity	0.05	-0.00	0.11	0.05	.061
	Contrast 2 → Symbolic Threat → Perceived Proximity	0.03	-0.03	0.08	0.03	.335
	Contrast 2 → Realistic Threat → Perceived Proximity	0.00	-0.04	0.05	0.00	.849
Component	Contrast 1 → Competition	-0.23	-0.44	-0.03	-0.11	<.05
	Competition → Perceived Proximity	-0.13	-0.25	$7.81^{e}-4$	-0.16	.051
	Contrast 1 → Symbolic Threat	0.01	-0.17	0.19	0.00	.902
	Symbolic Threat → Perceived Proximity	-0.06	-0.17	0.06	-0.06	.226
	Contrast 1 → Realistic Threat	-0.12	-0.31	0.07	-0.06	.226
	Realistic Threat → Perceived Proximity	-0.01	-0.14	0.12	-0.01	.848
	Contrast 2 → Competition	-0.42	-0.54	-0.30	-0.33	<.001
	Contrast 2 → Symbolic Threat	-0.48	-0.59	-0.38	-0.43	<.001
	Contrast 2 → Realistic Threat	-0.35	-0.46	-0.23	-0.30	<.001
Direct	Contrast 1 → Perceived Proximity	-0.46	-0.61	-0.31	-0.27	<.001
	Contrast 2 → Perceived Proximity	0.28	0.18	0.37	0.28	<.001
Total	Contrast 1 → Perceived Proximity	-0.43	-0.58	-0.28	-0.26	<.001

	Contrast 2 → Perceived Proximity	0.36	0.27	0.45	0.37	<.001

Table 6

Mediation Analyses on Intergroup Attitudes

Type	Effect	b	95% CI		β	p
			Lower	Upper		
Indirect	Contrast 1 (supplanting vs ideologically opposing) → Competition → Attitudes	0.70	-0.11	1.50	0.02	.090
	Contrast 1 → Symbolic Threat → Attitudes	-0.05	-0.80	0.71	-0.00	.902
	Contrast 1 → Realistic Threat → Attitudes	0.06	-0.23	0.35	0.00	.681
	Contrast 2 (allied vs other groups) → Competition → Attitudes	1.27	0.25	2.29	0.06	<.05
	Contrast 2 → Symbolic Threat → Attitudes	2.06	0.96	3.15	0.10	<.001
	Contrast 2 → Realistic Threat → Attitudes	0.18	-0.63	0.99	0.01	.663
Component	Contrast 1 → Competition	-0.23	-0.44	-0.03	-0.11	<.05
	Competition → Attitudes	-3.00	-5.27	-0.74	-0.18	<.01
	Contrast 1 → Symbolic Threat	0.01	-0.17	0.19	0.00	.902
	Symbolic Threat → Attitudes	-4.26	-6.33	-2.19	-0.23	<.001
	Contrast 1 → Realistic Threat	-0.12	-0.31	0.07	-0.06	.226
	Realistic Threat → Attitudes	-0.52	-2.85	1.81	-0.03	.662
	Contrast 2 → Competition	-0.42	-0.54	-0.30	-0.33	<.001
	Contrast 2 → Symbolic Threat	-0.48	-0.59	-0.38	-0.42	<.001
	Contrast 2 → Realistic Threat	-0.35	-0.46	-0.24	-0.30	<.001
Direct	Contrast 1 → Attitudes	-10.15	-12.79	-7.51	-0.28	<.001
	Contrast 2 → Attitudes	7.93	6.25	9.60	0.37	<.001

Total	Contrast 1 → Attitudes	-9.44	-12.37	-6.51	-0.26	<.001
	Contrast 2 → Attitudes	11.43	9.73	13.14	0.54	<.001

Table 7

Mediation Analyses on Cooperation Intentions

Type	Effect	b	95% CI		β	p
			Lower	Upper		
Indirect	Contrast 1 (supplanting vs ideologically opposing) → Competition → Cooperation Intentions	0.02	-0.01	0.05	0.01	0.161
	Contrast 1 → Symbolic Threat → Cooperation Intentions	-0.00	-0.07	0.06	-0.00	0.902
	Contrast 1 → Realistic Threat → Cooperation Intentions	-0.00	-0.02	0.01	-0.00	0.686
	Contrast 2 (allied vs other groups) → Competition → Cooperation Intentions	0.04	-0.00	0.09	0.04	0.081
	Contrast 2 → Symbolic Threat → Cooperation Intentions	0.18	0.12	0.24	0.16	< .001
	Contrast 2 → Realistic Threat → Cooperation Intentions	-0.01	-0.05	0.03	-0.01	0.669
Component	Contrast 1 → Competition	-0.23	-0.43	-0.03	-0.11	0.025
	Competition → Cooperation Intentions	-0.10	-0.21	0.01	-0.11	0.072
	Contrast 1 → Symbolic Threat	0.01	-0.17	0.19	0.00	0.902
	Symbolic Threat → Cooperation Intentions	-0.37	-0.47	-0.27	-0.38	< .001
	Contrast 1 → Realistic Threat	-0.12	-0.31	0.07	-0.06	0.226
	Realistic Threat → Cooperation Intentions	0.02	-0.09	0.14	0.02	0.668
	Contrast 2 → Competition	-0.42	-0.54	-0.30	-0.33	< .001
	Contrast 2 → Symbolic Threat	-0.48	-0.59	-0.38	-0.42	< .001
	Contrast 2 → Realistic Threat	-0.35	-0.46	-0.23	-0.30	< .001

Direct	Contrast 1 → Cooperation Intentions	-0.69	-0.81	-0.56	-0.36	< .001
	Contrast 2 → Cooperation Intentions	0.36	0.28	0.44	0.33	< .001
Total	Contrast 1 → Cooperation Intentions	-0.67	-0.82	-0.52	-0.36	< .001
	Contrast 2 → Cooperation Intentions	0.57	0.48	0.66	0.52	< .001

Table 8

Mediation Analyses on Perceived Warmth

Type	Effect	b	95% CI		β	p
			Lower	Upper		
Indirect	Contrast 1 (supplanting vs ideologically opposing) → Competition → Warmth	0.04	-0.00	0.09	0.02	0.071
	Contrast 1 → Symbolic Threat → Warmth	-0.00	-0.04	0.03	-0.00	0.902
	Contrast 1 → Realistic Threat → Warmth	-0.00	-0.02	0.01	-0.00	0.502
	Contrast 2 (allied vs other groups) → Competition → Warmth	0.08	0.02	0.13	0.07	0.005
	Contrast 2 → Symbolic Threat → Warmth	0.11	0.05	0.17	0.10	< .001
	Contrast 2 → Realistic Threat → Warmth	-0.02	-0.06	0.02	-0.01	0.424
Component	Contrast 1 → Competition	-0.23	-0.43	-0.03	-0.11	0.025
	Competition → Warmth	-0.18	-0.30	-0.07	-0.21	0.002
	Contrast 1 → Symbolic Threat	0.01	-0.17	0.19	0.00	0.902
	Symbolic Threat → Warmth	-0.23	-0.34	-0.13	-0.24	< .001
	Contrast 1 → Realistic Threat	-0.12	-0.31	0.07	-0.06	0.226
	Realistic Threat → Warmth	0.05	-0.07	0.17	0.05	0.420
	Contrast 2 → Competition	-0.42	-0.54	-0.30	-0.33	< .001
	Contrast 2 → Symbolic Threat	-0.48	-0.59	-0.38	-0.42	< .001

	Contrast 2 → Realistic Threat	-0.35	-0.46	-0.24	-0.30	< .001
Direct	Contrast 1 → Warmth	-0.42	-0.56	-0.28	-0.22	< .001
	Contrast 2 → Warmth	0.46	0.37	0.55	0.41	< .001
Total	Contrast 1 → Warmth	-0.38	-0.54	-0.23	-0.20	< .001
	Contrast 2 → Warmth	0.63	0.54	0.72	0.57	< .001

Table 9

Mediation Analyses on Perceived Competence

Type	Effect	b	95% CI		β	p
			Lower	Upper		
Indirect	Contrast 1 (supplanting vs ideologically opposing) → Competition → Competence	0.02	-0.01	0.05	0.01	0.180
	Contrast 1 → Symbolic Threat → Competence	-0.00	-0.04	0.03	-0.00	0.902
	Contrast 1 → Realistic Threat → Competence	-0.01	-0.04	0.01	-0.01	0.315
	Contrast 2 (allied vs other groups) → Competition → Competence	0.04	-0.01	0.09	0.04	0.103
	Contrast 2 → Symbolic Threat → Competence	0.09	0.04	0.15	0.10	< .001
	Contrast 2 → Realistic Threat → Competence	-0.04	-0.08	0.00	-0.04	0.084
Component	Contrast 1 → Competition	-0.23	-0.43	-0.03	-0.11	0.025
	Competition → Competence	-0.10	-0.21	0.02	-0.13	0.094
	Contrast 1 → Symbolic Threat	0.01	-0.17	0.19	0.00	0.902
	Symbolic Threat → Competence	-0.19	-0.30	-0.09	-0.23	< .001
	Contrast 1 → Realistic Threat	-0.12	-0.31	0.07	-0.06	0.226
	Realistic Threat → Competence	0.11	-0.10	0.22	0.13	0.072
	Contrast 2 → Competition	-0.42	-0.54	-0.30	-0.33	< .001

	Contrast 2 → Symbolic Threat	-0.48	-0.59	-0.38	-0.42	< .001
	Contrast 2 → Realistic Threat	-0.35	-0.46	-0.24	-0.30	< .001
Direct	Contrast 1 → Competence	-0.51	-0.64	-0.37	-0.31	< .001
	Contrast 2 → Competence	0.32	0.24	0.41	0.35	< .001
Total	Contrast 1 → Competence	-0.50	-0.63	-0.36	-0.31	< .001
	Contrast 2 → Competence	0.42	0.34	0.50	0.45	< .001

Study 3

Method

Measures

Confirmatory factor analysis

The factorial structure of the scale composed of items on competition over social recognition, symbolic, and realistic threats was satisfactory (Hu & Bentler, 1999), $\chi^2/df = 1.285$, CFI = .997, RMSEA = .0270 95% CI [.00, .0464], SRMR = .0153.

Results

As in Study 1 and Study 2, we preregistered hypotheses regarding how warm and competent the three types of groups would be perceived. As in Study 2, we hypothesised that a supplanting subgroup would be perceived as warmer and more competent than an ideologically opposing group, but colder and less competent than an allied subgroup**.** According to our hypotheses, and as in Studies 1 and 2, we found that a supplanting subgroup was perceived as warmer and more competent (μ_{warmth}= 4.46, SD_{warmth}= 1.26; $\mu_{competence}$=5.43, $SD_{competence}$=1.10) than an ideologically opposing group (μ_{warmth}=2.93, SD_{warmth}= 1.46; $\mu_{competence}$= 4.11, $SD_{competence}$= 1.45), but colder and less competent than an allied subgroup (μ_{warmth}= 5.68, SD_{warmth}= 1.12; $\mu_{competence}$= 5.92, $SD_{competence}$= 1.01). Statistical details can be found in Table 10.

Table 10

Impact of the Type of Group on Perceived Warmth and Competence

	Contrasts									
	Contrast 1 – Supplanting vs Ideologically Opposing Group					Contrast 2 – Allied Subgroup vs Other Groups				
	b	CI Min	CI Max	t-value	*p-value*	b	CI Min	CI Max	t-value	*p-value*
Perceived Warmth	-0.77	-0.92	-0.61	-9.67	<.001	0.66	0.57	0.75	14.21	<.001
Perceived Competence	-0.66	-0.81	-0.52	-8.93	<.001	0.38	0.30	0.47	8.76	<.001

In Table 11, we report Games-Howell post-hoc comparisons to attest to the difference between each type of group on our outcome variables, especially between a supplanting subgroup and an allied subgroup.

Table 11

Post Hoc Comparisons Study 3

	Supplanting vs Allied		*Supplanting vs Ideologically Opposing*		*Allied vs Ideologically Opposing*	
	t-value	*P-value*	t-value	*p-value*	t-value	*p-value*
Perceived Proximity	-5.64	<.001	8.17	<.001	14.46	<.001
Intergroup Attitude	-7.67	<.001	10.4	<.001	20.1	<.001
Cooperation Intentions	-2.68	<.05	12.7	<.001	16.2	<.001
Perceived Warmth	-8.10	<.001	9.23	<.001	17.41	<.001
Perceived Competence	-3.63	.001	8.48	<.001	11.97	<.001
Perceived Competition over Prototypicality	10.3	<.001	0.59	0.825	-9.88	<.001
Perceived Symbolic Threat	8.72	<.001	-1.05	0.546	-10.33	<.001
Perceived Realistic Threat	8.33	<.001	0.74	0.741	-7.94	<.001

Regarding our mediation hypotheses, it appears that symbolic threat mediated the relations between C2 and perceived warmth as well as between C2 and perceived competence. This suggests that an allied subgroup was perceived as warmer and more competent than the two other groups and that this difference is mediated by less perception of symbolic threat. These results corroborate our findings, suggesting that a supplanting subgroup may also trigger ideological conflicts within social movements.

Figure 3

Mediation Analyses on Perceived Warmth in Study 3

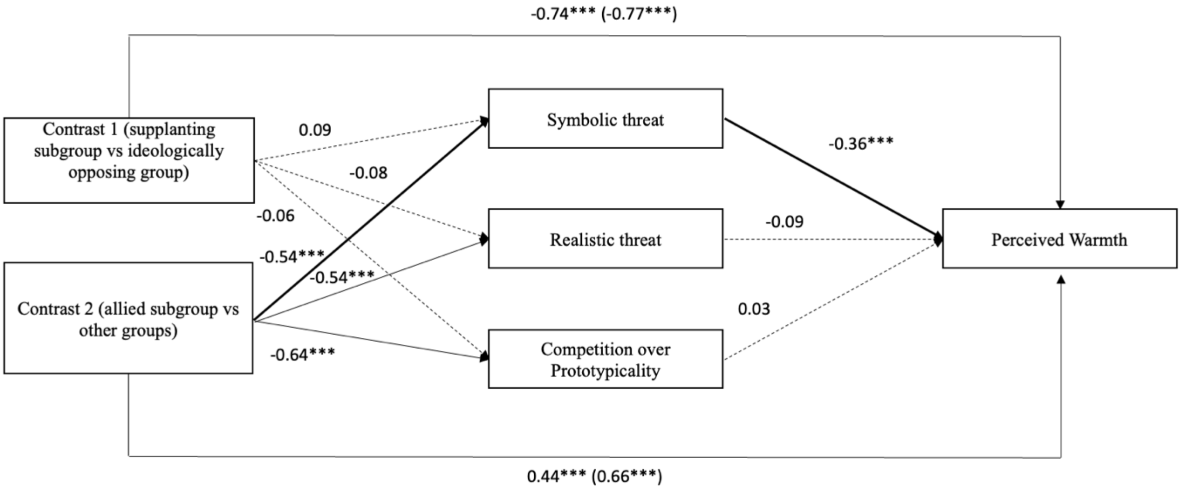

Note. A full arrow indicates that the path is significant. An interrupted arrow indicates that the path is not significant.

Figure 4

Mediation Analyses on Perceived Competence in Study 3

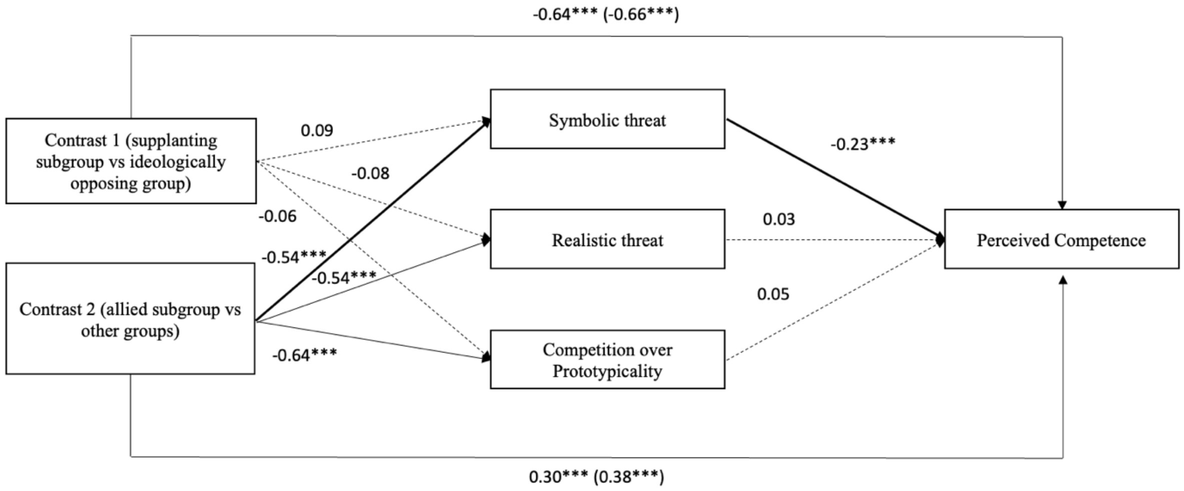

Note. A full arrow indicates that the path is significant. An interrupted arrow indicates that the path is not significant.

In Tables 12 to 16, we report the detailed statistics of mediation analyses for each dependent variable.

Table 12

Mediation Analyses on Perceived Proximity

Type	Effect	b	95% CI Lower	95% CI Upper	β	p
Indirect	Contrast 1 (supplanting vs ideologically opposing) → Competition → Perceived Proximity	0.00	-0.01	0.02	0.00	0.562
	Contrast 1 → Symbolic Threat → Perceived Proximity	-0.01	-0.04	0.01	-0.01	0.299
	Contrast 1 → Realistic Threat → Perceived Proximity	-3.93e−4	-0.01	0.01	-2.51e−4	0.920
	Contrast 2 (allied vs other groups) → Competition → Perceived Proximity	0.06	-0.01	0.13	0.07	0.088
	Contrast 2 → Symbolic Threat → Perceived Proximity	0.09	0.04	0.14	0.10	0.001
	Contrast 2 → Realistic Threat → Perceived Proximity	-0.00	-0.06	0.05	-0.00	0.920
Component	Contrast 1 → Competition	-0.06	-0.25	0.13	-0.03	0.539
	Competition → Perceived Proximity	-0.09	-0.20	0.01	-0.13	0.084
	Contrast 1 → Symbolic Threat	0.09	-0.07	0.26	0.048	0.276
	Symbolic Threat → Perceived Proximity	-0.17	-0.26	-0.07	-0.20	< .001
	Contrast 1 → Realistic Threat	-0.08	-0.27	0.12	-0.03	0.445
	Realistic Threat → Perceived Proximity	0.00	-0.09	0.10	0.01	0.920
	Contrast 2 → Competition	-0.64	-0.75	-0.53	-0.50	< .001
	Contrast 2 → Symbolic Threat	-0.54	-0.63	-0.44	-0.48	< .001
	Contrast 2 → Realistic Threat	-0.54	-0.65	-0.42	-0.42	< .001
Direct	Contrast 1 → Perceived Proximity	-0.53	-0.65	-0.41	-0.34	< .001
	Contrast 2 → Perceived Proximity	0.28	0.19	0.36	0.30	< .001
Total	Contrast 1 → Perceived Proximity	-0.54	-0.67	-0.42	-0.35	< .001

	Contrast 2 → Perceived Proximity	0.42	0.35	0.50	0.46	< .001

Table 13

Mediation Analyses on Intergroup Attitudes

Type	Effect	b	95% CI Lower	95% CI Upper	β	p
Indirect	Contrast 1 (supplanting vs ideologically opposing) → Competition → Intergroup Attitudes	-0.63	-1.77	0.51	-0.02	0.281
	Contrast 1 → Symbolic Threat → Intergroup Attitudes	0.04	-0.14	0.23	0.00	0.651
	Contrast 1 → Realistic Threat → Intergroup Attitudes	0.12	-0.29	0.53	0.00	0.558
	Contrast 2 (allied vs other groups) → Competition → Intergroup Attitudes	3.64	2.44	4.84	0.16	< .001
	Contrast 2 → Symbolic Threat → Intergroup Attitudes	0.30	-0.75	1.35	0.01	0.576
	Contrast 2 → Realistic Threat → Intergroup Attitudes	1.33	-0.03	2.69	0.06	0.055
Component	Contrast 1 → Competition	0.09	-0.07	0.26	0.05	0.276
	Competition → Intergroup Attitudes	-6.78	-8.65	-4.92	-0.34	< .001
	Contrast 1 → Symbolic Threat	-0.08	-0.27	0.12	-0.03	0.445
	Symbolic Threat → Intergroup Attitudes	-0.55	-2.50	1.39	-0.03	0.575
	Contrast 1 → Realistic Threat	-0.06	-0.25	0.13	-0.03	0.539
	Realistic Threat → Intergroup Attitudes	-2.07	-4.15	0.02	-0.12	0.052
	Contrast 2 → Competition	-0.54	-0.63	-0.44	-0.48	< .001
	Contrast 2 → Symbolic Threat	-0.54	-0.65	-0.42	-0.42	< .001
	Contrast 2 → Realistic Threat	-0.64	-0.75	-0.53	-0.50	< .001

Direct	Contrast 1 → Intergroup Attitudes	-15.20	-17.52	-12.88	-0.39	< .001
	Contrast 2 → Intergroup Attitudes	7.18	5.59	8.77	0.32	< .001
Total	Contrast 1 → Intergroup Attitudes	-15.66	-18.41	-12.91	-0.41	< .001
	Contrast 2 → Intergroup Attitudes	12.45	10.84	14.07	0.55	< .001

Table 14

Mediation Analyses on Cooperation Intentions

Type	Effect	b	95% CI		β	p
			Lower	Upper		
Indirect	Contrast 1 (supplanting vs ideologically opposing) → Competition → Cooperation Intentions	-0.03	-0.09	0.03	-0.02	0.281
	Contrast 1 → Symbolic Threat → Cooperation Intentions	0.00	-0.01	0.01	6.39e-4	0.780
	Contrast 1 → Realistic Threat → Cooperation Intentions	0.00	-0.01	0.01	0.00	0.653
	Contrast 2 (allied vs other groups) → Competition → Cooperation Intentions	0.19	0.13	0.25	0.18	< .001
	Contrast 2 → Symbolic Threat → Cooperation Intentions	0.01	-0.04	0.06	0.01	0.764
	Contrast 2 → Realistic Threat → Cooperation Intentions	0.02	-0.04	0.09	0.02	0.510
Component	Contrast 1 → Competition	0.09	-0.07	0.26	0.05	0.276
	Competition → Cooperation Intentions	-0.35	-0.44	-0.26	-0.39	< .001
	Contrast 1 → Symbolic Threat	-0.08	-0.27	0.12	-0.03	0.445
	Symbolic Threat → Cooperation Intentions	-0.01	-0.11	0.08	-0.02	0.764
	Contrast 1 → Realistic Threat	-0.06	-0.25	0.13	-0.03	0.539
	Realistic Threat → Cooperation Intentions	-0.03	-0.14	0.07	-0.04	0.509
	Contrast 2 → Competition	-0.54	-0.63	-0.44	-0.48	< .001
	Contrast 2 → Symbolic Threat	-0.54	-0.65	-0.42	-0.43	< .001
	Contrast 2 → Realistic Threat	-0.64	-0.75	-0.53	-0.50	< .001
Direct	Contrast 1 → Cooperation Intentions	-0.85	-0.96	-0.73	-0.49	< .001
	Contrast 2 → Cooperation Intentions	0.19	0.11	0.27	0.19	< .001
Total	Contrast 1 → Cooperation Intentions	-0.87	-1.00	-0.74	-0.50	< .001

	Contrast 2 → Cooperation Intentions	0.41	0.33	0.49	0.40	< .001

Table 15

Mediation Analyses on Perceived Warmth

Type	Effect	b	95% CI		β	p
			Lower	Upper		
Indirect	Contrast 1 (supplanting vs ideologically opposing) → Competition → Perceived Warmth	-0.00	-0.01	0.01	-8.18e−4	0.717
	Contrast 1 → Symbolic Threat → Perceived Warmth	-0.03	-0.09	0.03	-0.02	0.283
	Contrast 1 → Realistic Threat → Perceived Warmth	0.01	-0.01	0.02	0.00	0.499
	Contrast 2 (allied vs other groups) → Competition → Perceived Warmth	-0.02	-0.10	0.06	-0.01	0.653
	Contrast 2 → Symbolic Threat → Perceived Warmth	0.19	0.12	0.26	0.16	< .001
	Contrast 2 → Realistic Threat → Perceived Warmth	0.05	-0.02	0.11	0.04	0.152
Component	Contrast 1 → Competition	-0.06	-0.25	0.13	-0.03	0.539
	Competition → Perceived Warmth	0.03	-0.10	0.15	0.03	0.653
	Contrast 1 → Symbolic Threat	0.09	-0.07	0.26	0.05	0.276
	Symbolic Threat → Perceived Warmth	-0.36	-0.48	-0.25	-0.33	< .001
	Contrast 1 → Realistic Threat	-0.08	-0.27	0.12	-0.03	0.445
	Realistic Threat → Perceived Warmth	-0.09	-0.20	0.03	-0.09	0.147
	Contrast 2 → Competition	-0.64	-0.75	-0.53	-0.50	< .001
	Contrast 2 → Symbolic Threat	-0.54	-0.63	-0.44	-0.48	< .001
	Contrast 2 → Realistic Threat	-0.54	-0.65	-0.42	-0.42	< .001
Direct	Contrast 1 → Perceived Warmth	-0.74	-0.88	-0.60	-0.35	< .001
	Contrast 2 → Perceived Warmth	0.44	0.34	0.53	0.36	< .001

Total	Contrast 1 → Perceived Warmth	-0.77	-0.92	-0.61	-0.37	< .001
	Contrast 2 → Perceived Warmth	0.66	0.57	0.75	0.54	< .001

Table 16

Mediation Analyses on Perceived Competence

Type	Effect	b	95% CI		β	p
			Lower	Upper		
Indirect	Contrast 1 (supplanting vs ideologically opposing) → Competition → Perceived Competence	-0.00	-0.01	0.09	-0.00	0.643
	Contrast 1 → Symbolic Threat → Perceived Competence	-0.02	-0.06	0.02	-0.01	0.294
	Contrast 1 → Realistic Threat → Perceived Competence	-0.00	-0.01	0.01	-0.00	0.665
	Contrast 2 (allied vs other groups) → Competition → Perceived Competence	-0.03	-0.11	0.05	-0.03	0.482
	Contrast 2 → Symbolic Threat → Perceived Competence	0.12	0.06	0.19	0.12	< .001
	Contrast 2 → Realistic Threat → Perceived Competence	-0.02	-0.08	0.05	-0.02	0.600
Component	Contrast 1 → Competition	-0.06	-0.25	0.13	-0.03	0.539
	Competition → Perceived Competence	0.05	-0.08	0.17	0.06	0.482
	Contrast 1 → Symbolic Threat	0.09	-0.07	0.26	0.05	0.276
	Symbolic Threat → Perceived Competence	-0.23	-0.34	-0.11	-0.25	< .001
	Contrast 1 → Realistic Threat	-0.08	-0.27	0.12	-0.03	0.445
	Realistic Threat → Perceived Competence	0.03	-0.09	0.15	0.04	0.600
	Contrast 2 → Competition	-0.64	-0.75	-0.53	-0.50	< .001
	Contrast 2 → Symbolic Threat	-0.54	-0.63	-0.44	-0.48	< .001
	Contrast 2 → Realistic Threat	-0.54	-0.65	-0.42	-0.42	< .001
Direct	Contrast 1 → Perceived Competence	-0.64	-0.78	-0.49	-0.36	< .001
	Contrast 2 → Perceived Competence	0.30	0.21	0.40	0.30	< .001

Total	Contrast 1 → Perceived Competence	-0.66	-0.81	-0.52	-0.38	< .001
	Contrast 2 → Perceived Competence	0.38	0.30	0.47	0.37	< .001

Materials

Experimental Material: Studies 1 and 2

Introduction: French Version (Studies 1-2)

“ Vlurville est une ville de 700.000 habitant·e·s. Capitale de Vlurland, elle accueille en son sein de nombreux mouvements sociaux luttant pour des causes diverses. Ainsi, il n’est pas rare que les plateaux télés et les émissions de radio relatent des manifestations ou d’autres actions organisées par les mouvements. Citoyen·ne de la ville depuis toujours, vous militez pour une cause au sein d’une association qui s’inscrit dans la lutte depuis 20 ans. L’association parvient souvent à attirer l’attention des pouvoirs décisionnaires et du grand public, notamment via des apparitions à la télévision et à la radio. En outre, les actions que vous organisez ont toujours eu un beau succès. Vos manifestations attirent entre 10.000 et 50.000 participant·e·s et vos pétitions recueillent de nombreuses signatures. Votre association est très connue dans le milieu et fait souvent office de référence, tant par les autres activistes que par les personnes ne luttant pas pour la cause. Votre implication au sein de l’association est importante et vous vous définissez comme un·e activiste. Vous participez aux réunions, aux actions, et vous n’hésitez pas à continuer votre lutte dans la sphère privée et professionnelle à travers des gestes de la vie quotidienne et des débats endiablés avec votre entourage. Cette cause est importante pour vous et votre implication au sein de l’association vous tient à cœur. »

Après avoir lu la description, prenez un temps pour imaginer et décrire par écrit à quoi ressemble votre quotidien de militant·e à Vlurville. Pour quelle cause militez-vous ? Que faites-vous dans le cadre de la lutte ? Cela vous prend-il beaucoup de temps ? Prenez votre temps, cette première partie du questionnaire est la plus longue mais est essentielle pour le bon déroulé de l'étude.

Introduction: English Translation (Studies 1-2)

«Vlurville is a city of 700,000 citizens. As the capital of Vlurland, it is home to many social movements struggling for various causes. Thus, it is not uncommon for TV and radio programs to report on demonstrations or other actions organized by the movements. As a lifelong citizen of the city, you are an activist and a part of an association that has been involved in the struggle for 20 years. The association often succeeds in attracting the attention of decision-makers and the general public, notably through appearances on television and radio. Moreover, the actions you organize have always been very successful. Your demonstrations attract between 10,000 and 50,000 participants and your petitions collect numerous signatures. Your association is very well known in the field and is often used as a reference, both by other activists and by people who do not fight for the cause. Your involvement in the association is important and you define yourself as an activist. You participate in meetings, actions, and you do not hesitate to continue your struggle in the private and professional sphere through everyday gestures and heated debates with your entourage. This cause is important to you and your involvement in the association is important to you. »

After reading the description, take a moment to imagine and describe in writing what your daily life is like as an activist in Vlurville. What's your cause? What do you do as part of the struggle? Does it take up a lot of your time? Take your time: this first part of the questionnaire is the longest, but it's essential for the study to run smoothly.

Supplanting subgroup: French Version (Studies 1-2)

« Après de nombreuses années passées au sein de la lutte, vous constatez que le mouvement en faveur de votre cause prend de l'ampleur. De nouveaux groupes de militant·e·s rejoignent la lutte et les actions se multiplient. Depuis quelques mois, un nouveau groupe en particulier semble attirer beaucoup d'attention de la part des citoyen·ne·s, ainsi que des médias. En effet, il n'est pas rare d'entendre des membres de ce nouveau groupe à la radio,

dans des émissions de débat, ou de les voir s'exprimer à la télévision. En outre, ils développent leur lutte sur les réseaux sociaux et ont beaucoup de succès. Ils semblent mettre en place des stratégies de communication plus récentes qui démocratisent la lutte et leur apporte beaucoup d'attention, notamment auprès du jeune public. Ce nouveau groupe lutte pour la même cause que vous, mais vous remarquez que les membres de votre groupe sont de moins en moins invités sur les plateaux et à la radio, vous ne faites plus autant d'interviews et d'interventions qu'avant. De plus, vos actions ont moins de visibilité depuis l'arrivée du nouveau groupe, et vous avez l'impression de ne plus être reconnus comme les acteurs et actrices phares de la lutte. Vous vous reconnaissez moins dans les actions politiques organisées par ce groupe qui a, en plus, parfois tendance à critiquer vos projets et vos stratégies.

Prenez désormais un instant pour imaginer et écrire le nom (fictif ou non) que porte le groupe présenté ci-dessus ainsi que pour décrire ses actions, ses comportements envers votre groupe, ainsi que votre ressenti. Nous vous demanderons ensuite de répondre à des questions en ayant en tête le groupe que nous venons d'introduire. »

Supplanting subgroup: English Translation (Studies 1-2)

After many years in the struggle, you see that the movement for your cause is growing. New groups of activists are joining the struggle and actions are multiplying. In the last few months, one new group in particular seems to be attracting a lot of attention from citizens, as well as from the media. Indeed, it is not uncommon to hear members of this new group on the radio, in talk shows, or to see them speaking on television. Moreover, they develop their struggle on social networks and are very successful. They seem to implement more recent communication strategies that democratize the struggle, especially with the young public. This new group fights for the same cause as you, but you notice that the members of your group are less invited on TV and radio shows, you don't do as many

interviews and interventions as before. Moreover, your actions are less successful since the arrival of the new group, and you feel you are no longer recognized as the central figures of the struggle. You feel less connected to the political actions organized by this group, which, on top of that, sometimes tends to criticize your projects and strategies.

Now take a moment to imagine and write down the name (fictitious or not) of the group presented above, as well as to describe its actions, its behaviors towards your groups. How do you feel about it ? We will then ask you to answer questions with the group we have just introduced in mind. »

Allied subgroup: French Version (Studies 1-2)

“Après de nombreuses années passées au sein de la lutte, vous constatez que le mouvement en faveur de votre cause prend de l'ampleur. De nouveaux groupes de militant·e·s rejoignent la lutte et les actions se multiplient. Depuis quelques mois, un nouveau groupe en particulier semble attirer beaucoup d'attention de la part des citoyen·ne·s, ainsi que des médias. En effet, il n'est pas rare d'entendre des membres de ce nouveau groupe à la radio, dans des émissions de débat, ou de les voir s'exprimer à la télévision. En outre, ils développent leur lutte sur les réseaux sociaux et ont beaucoup de succès. Ils semblent mettre en place des stratégies de communication qui démocratisent la lutte et leur apporte beaucoup d'attention, notamment auprès du jeune public. Ce nouveau groupe lutte pour la même cause que vous, et prend toujours soin de mettre en avant les autres membres de la lutte, dont votre groupe. Ils ont comme volonté d’établir une stratégie commune et de veiller à ce que chacun et chacune se sente écouté·e et entendu·e. Ils vous ont d’ailleurs permis de rejoindre à votre tour les réseaux sociaux et vous partagez vos idées d’actions et de stratégie. Les membres du groupe relaient les informations vous concernant et n’hésitent pas à vous défendre quand cela est nécessaire. A l’inverse, les membres de votre groupe participent également à leurs actions et vous faites tous preuve de beaucoup de solidarité au sein de la lutte.

Prenez désormais un instant pour imaginer le nom (fictif ou non) que porte le groupe présenté ci-dessus ainsi que ses actions, ses comportements envers votre groupe, ainsi que votre ressenti. Nous vous demanderons ensuite de répondre à des questions en ayant en tête le groupe que nous venons d'introduire. »

Allied Subgroup : English Translation (Studies 1-2)

«After many years in the struggle, you see that the movement for your cause is growing. New groups of activists are joining the struggle and actions are multiplying. In the last few months, one new group in particular seems to be attracting a lot of attention from citizens, as well as from the media. Indeed, it is not uncommon to hear members of this new group on the radio, in talk shows, or to see them speaking on television. Moreover, they develop their struggle on social networks and are very successful. They seem to set up communication strategies that democratize the struggle and bring them a lot of attention, especially among the young public. This new group fights for the same cause as you, and always takes care to put forward the other members of the struggle, including your group. They want to establish a common strategy and to make sure that everyone feels heard and listened to. They have also allowed you to join the social networks and share your ideas for actions and strategies. The members of the group relay information about you and do not hesitate to defend you when necessary. Conversely, your group members also participate in their actions and you all show a lot of solidarity within the struggle.

Now take a moment to imagine the name (fictitious or not) of the group presented above, as well as its actions, its behavior towards your group. How do you feel about it? We will then ask you to answer questions with the group we have just introduced in mind. »

Ideologically Opposing Group: French Version (Studies 1-2)

« Après de nombreuses années passées au sein de la lutte, vous constatez que le mouvement en faveur de votre cause prend de l'ampleur. De nouveaux groupes de militant·e·s

rejoignent la lutte et les actions se multiplient. Cependant, tout le monde n'est pas d'accord avec les idées défendues par votre mouvement. En effet, un groupe de citoyens et citoyennes se définissant comme activistes organisent des actions qui défendent une idéologie à l'opposé de la vôtre. En outre, il n'est pas rare de les entendre à la radio ou dans des émissions de débat, au même titre que les membres de votre mouvement. Les médias vous appellent souvent pour débattre l'un face à l'autre et vous devez faire face à des critiques qui vont à l'opposé de vos idées. Vos échanges sont souvent houleux et vous êtes en profond désaccord sur votre vision de la société et de la cause que vous défendez. Ce groupe a une visibilité assez similaire à la vôtre et ses membres sont actifs dans les médias et sur les réseaux sociaux.

Prenez désormais un instant pour imaginer le nom (fictif ou non) que porte le groupe présenté ci-dessus ainsi que ses actions, ses comportements envers votre groupe, ainsi que votre ressenti. Nous vous demanderons ensuite de répondre à des questions en ayant en tête le groupe que nous venons d'introduire. »

Ideologically Opposing Group: English Translation (Studies 1-2)

«After many years in the struggle, you see that the movement for your cause is growing. New groups of activists are joining the struggle and actions are multiplying. However, not everyone agrees with the ideas defended by your movement. Indeed, a group of citizens defining themselves as activists organize actions that defend an ideology opposite to yours. Moreover, it is not uncommon to hear them on the radio or on talk shows, just like the members of your movement. The media often calls you to debate each other and you have to deal with criticism that goes against your ideas. Your exchanges are often heated and you disagree deeply on your vision of society and the cause you defend.

Now take a moment to imagine the name (fictitious or not) of the group presented above, as well as its actions and behaviors towards your group. How do you feel about it ? We will then ask you to answer some questions with the group we have just introduced in mind. »

Experimental Material: Study 3

Introduction: English Version (Study 3)

«Vlurville is a city of 700,000 citizens. As the capital of Vlurland, it is home to many social movements struggling for various causes. Thus, it is not uncommon for TV and radio programs to report on demonstrations or other actions organized by the movements. As a lifelong citizen of the city, you are an activist and a part of an association that has been involved in the struggle for 20 years. The association often succeeds in attracting the attention of decision-makers and the general public, notably through appearances on television and radio. Moreover, the actions you organize have always been very successful. Your demonstrations attract between 10,000 and 50,000 participants and your petitions collect numerous signatures. Your association is very well known in the field and is often used as a reference, both by other activists and by people who do not fight for the cause. Your involvement in the association is important and you define yourself as an activist. You participate in meetings, actions, and you do not hesitate to continue your struggle in the private and professional sphere through everyday gestures and heated debates with your entourage. This cause is important to you and your involvement in the association is important to you. »

Supplanting Subgroup: English Version (Study 3)

Some changes were made between the versions of the vignette presenting a supplanting subgroup used in Studies 1 & 2 and that used in Study 3 to decrease the risk of experimental demand effects. First, regarding the distinctiveness threat of a supplanting subgroup, the vignette in Studies 1 & 2 mentioned "You recognize yourself less in the

political actions organized by this group". Second, regarding the perceived ability of supplanting subgroup to attract social recognition, it mentioned "You feel that you are no longer recognized as the key players in the struggle". We decided to remove these two sentences from the vignette used in Study 3.

«After many years in the struggle, you see that the movement for your cause is growing. New groups of activists are joining the struggle and actions are multiplying. In the last few months, one new group in particular seems to be attracting a lot of attention from citizens, as well as from the media. Indeed, it is not uncommon to hear members of this new group on the radio, in talk shows, or to see them speaking on television. Moreover, they develop their struggle on social networks and are very successful. They seem to implement more recent communication strategies that democratize the struggle, especially with the young public. This new group fights for the same cause as you, but you notice that the members of your group are less invited on TV and radio shows, you don't do as many interviews and interventions as before. Moreover, your actions are less successful since the arrival of the new group. In addition, this group sometimes tends to criticize your projects and strategies. Now take a moment to imagine and write down the name (fictitious or not) of the group presented above, as well as to describe its actions, its behaviors towards your groups. How do you feel about it ? We will then ask you to answer questions with the group we have just introduced in mind. »

Allied Subgroup: English Version (Study 3)

«After many years in the struggle, you see that the movement for your cause is growing. New groups of activists are joining the struggle and actions are multiplying. In the last few months, one new group in particular seems to be attracting a lot of attention from citizens, as well as from the media. Indeed, it is not uncommon to hear members of this new group on the radio, in talk shows, or to see them speaking on television. Moreover, they develop their struggle on social networks and are very successful. They seem to set up

communication strategies that democratize the struggle and bring them a lot of attention, especially among the young public. This new group fights for the same cause as you, and always takes care to put forward the other members of the struggle, including your group. They want to establish a common strategy and to make sure that everyone feels heard and listened to. They have also allowed you to join the social networks and share your ideas for actions and strategies. The members of the group relay information about you and do not hesitate to defend you when necessary. Conversely, your group members also participate in their actions and you all show a lot of solidarity within the struggle. Now take a moment to imagine the name (fictitious or not) of the group presented above, as well as its actions, its behavior towards your group. How do you feel about it? We will then ask you to answer questions with the group we have just introduced in mind. »

Ideologically Opposing group: English Version (Study 3)

«After many years in the struggle, you see that the movement for your cause is growing. New groups of activists are joining the struggle and actions are multiplying. However, not everyone agrees with the ideas defended by your movement. Indeed, a group of citizens defining themselves as activists organize actions that defend an ideology opposite to yours. Moreover, it is not uncommon to hear them on the radio or on talk shows, just like the members of your movement. The media often calls you to debate each other and you have to deal with criticism that goes against your ideas. Your exchanges are often heated and you disagree deeply on your vision of society and the cause you defend. Now take a moment to imagine the name (fictitious or not) of the group presented above, as well as its actions and behaviors towards your group. How do you feel about it ? We will then ask you to answer some questions with the group we have just introduced in mind. »

Measures

Competition over Social Recognition as the Prototypical Figure of the Movement (Studies 2-3)

«With the group just presented in mind, please indicate the extent to which you agree with the statements below. »

a) I'm afraid that the focus on the members of this group will take away the recognition that my group deserves as a representative of the struggle.
b) The media is paying so much attention to this new group that I fear there is no more attention for ours.
c) I feel that the actions of my group might be forgotten because all the attention is attributed to the actions of this group.
d) Since the arrival of this group, I feel that my group is no longer seen as a central figure in the struggle.
e) I am concerned that the actions taken by my group within the movement are not being recognized because all the recognition is being captured by this new group.

Symbolic Threat (Studies 2-3)

«With the group just presented in mind, please indicate the extent to which you agree with the statements below. » (1-totally disagree to 7-totally agree)

a) I fear that my group's culture will be discredited by the presented group.
b) I think the arrival of this group makes the cause more enjoyable to defend. (Reversed item)
c) The values and ideology of my group might be tainted by the presented group.

Realistic Threat (Studies 2-3)

« With the group presented to you just now in mind, please indicate the extent to which you agree with the statements below. » (1-totally disagree to 7-totally agree)

a) I am afraid that the members of the presented group will occupy the positions of responsibility normally occupied by the members of my group.
b) The presented group could take the subsidies normally granted to my group and that worries me.
c) Since the arrival of this group, I have the impression that the authorities could give us less material support for our actions.

Perceived Proximity

«Look at the five figures below. The largest circle represents your group while the smallest represents the group presented at the beginning of your study. Where do you position the group presented in relation to your group? »

Based on Swann et al., 2009

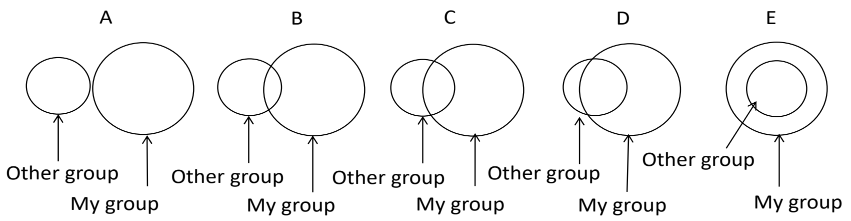

Intergroup Attitude

«Please indicate your overall feeling about the group presented. 0 represents a very negative feeling and 100 represents a very positive feeling. 50 represents a neutral feeling for the group presented. »

Perceived Warmth and Perceived Competence

«The following questions are about your impressions of the group you were introduced to. In your opinion, are the members of this group... » 1-totally disagree to 7-totally agree

a) ... friendly ?
b) ... caring for others ?
c) ... trustworthy ?
d) ... warm ?
e) ... good natured ?
f) ... sincere ?
g) ... competent ?
h) ... confident ?
i) ... capable ?
j) ... effective ?
k) ... smart ?
l) ... skilled ?

Cooperation Intentions

«To what extent do you intend to... »

a) ... work on a common project with them?
b) ... share the material resources of your group with them?
c) ... create a new common movement with them?
d) ... prevent them to intervene in the media (TV, radio, internet, etc.)? (Reversed item)
e) ... refuse to associate them with the general struggle? (Reversed item)
f) ... enter into open conflict with them? (Reversed item)
g) ... have a constructive and respectful debate with them?
h) ... support them in their actions?

Attentioncheck and seriousness check

The attention check item is a part of the perceived warmth and competence scale, required participants to provide a specific answer (rated 4 on a Likert scale) to verify they were not automated bots (Bowling et al., 2016). The second item, placed at the end of the survey, inquired whether participants approached the study seriously and granted permission for the use of their responses (Aust et al., 2013).
